# High-throughput *ab initio* calculations on dielectric constant and band gap of non-oxide dielectrics

**DOI:** 10.1038/s41598-018-33095-6

**Published:** 2018-10-04

**Authors:** Miso Lee, Yong Youn, Kanghoon Yim, Seungwu Han

**Affiliations:** 1 Department of Materials Science and Engineering and Research Institute of Advanced Materials, Seoul National University, Seoul, 08826 Korea; 2 Korea Institute of Energy Research, Daejeon, 34129 Korea

## Abstract

High-*k* dielectrics, materials having a large band gap (*E*_g_) and high dielectric constant (*k*) simultaneously, constitute critical components in microelectronic devices. Because of the inverse relationship between *E*_g_ and *k*, materials with large values in both properties are rare. Therefore, massive databases on *E*_g_ and *k* will be useful in identifying optimal high-*k* materials. While experimental and theoretical data on *E*_g_ and *k* of oxides are accumulating, corresponding information is scarce for non-oxide dielectrics with anions such as C, N, F, P, S, and Cl. To identify promising high-*k* dielectrics among these material groups, we screen 869 compounds of binary carbides, nitrides, sulfides, phosphides, chlorides, and fluorides, through automated *ab initio* calculations. Among these compounds, fluorides exhibit an *E*_g_-*k* relation that is comparable to that of oxides. By further screening over ternary fluorides, we identify fluorides such as BiF_3_, LaF_3_, and BaBeF_4_ that could serve as useful high-*k* dielectrics.

## Introduction

Continuous scaling of silicon-based transistors has led the rapid growth of the semiconductor industry over the last 40 years. During this period, high-quality interfaces between the Si substrate and SiO_2_ gate dielectrics facilitated the steady downscaling of Si devices, which accelerated the operation speed while reducing the power consumption^1^. However, as the thickness of SiO_2_ is decreased to less than a few nanometers, the traditional fabrication process faced with significant leakage currents that originate from tunneling through ultrathin SiO_2_ dielectrics^1–3^. This was resolved by incorporating high dielectric constant (high-*k*) materials such as HfO_2_ and ZrO_2_^3–8^. These high-*k* oxides can reduce leakage currents by increasing the physical thickness of insulating layers while enhancing capacitive coupling between the channel layer and gate electrode.

Currently, the rapid expansion of mobile devices and high-performance computing markets are driving further development of transistors towards higher performance and lower power consumption. This in turn necessitates dielectric materials with higher-*k* than those of HfO_2_ or ZrO_2_; according to the International Roadmap for Devices and Systems (IRDS)^9^, dielectrics with *k* of 50~100 will be required in transistors or capacitors by 2024. Among the oxides, rutile TiO_2_ or SrTiO_3_ with *k* >100 are attracting interests as next-generation gate dielectrics but their small band gaps cause significant leakage currents^7,10–13^. On the other hand, new channel materials such as Ge, InSb and InGaAs are considered for the next-generation semiconducting devices because intrinsic carrier mobilities are higher in these materials than in Si^9^. However, the interface between these materials and oxides, for instance Ge/GeO_2_ and InGaAs/HfO_2_, are more defective than the Si/SiO_2_ interface, degrading the carrier mobility in actual devices^14–17^. The foregoing discussions indicate that a more diverse library of high-*k* materials will be beneficial in coping with the challenges in next-generation semiconducting devices. In particular, non-oxide dielectrics may provide solutions to issues that occur with conventional oxide dielectrics. For example, a recent study showed that CaF_2_ is superior to Al_2_O_3_ as a gate dielectric layer in the p-GaN device by reducing interface trap densities^18^. It was also reported that CaF_2_ forms a stable interface with the GaAs substrate^19^. Therefore, the property database of dielectric constants and band gaps (*E*_g_’s) covering both oxides and non-oxides will be useful in selecting optimal high-*k* dielectrics.

Considering the huge material space of known dielectrics, it is not feasible to build a big database on *E*_g_ and *k* experimentally. Recently, owing to development of the density-functional theory (DFT) and exponentially growing computational speed, it becomes possible to conduct massive calculations on dielectric and electronic properties of crystals. In a previous study^20^, we carried out high-throughput DFT screening over ~1,800 oxides and identified new candidate high-*k* oxides such as c-BeO whose figure of merit far exceeding that of industry-standard HfO_2_. However, discussions in the above imply that non-oxide dielectrics would be also valuable in view of exploiting diverse chemistry. Recently, Petousis *et al*. performed high-throughput screening on ~1,000 inorganic compounds including both oxide and non-oxide compounds^21^. However, the search space in ref.^21^. was limited to stable or metastable phases with hull energies in the phase diagram less than 20 meV/atom, and relatively small primitive cells containing less than 20 atoms. However, such restrictive conditions could miss promising high-*k* materials. For example, the industry-standard HfO_2_ thin film includes a significant portion of tetragonal or cubic phases that exhibit high *k* values^22^. However, these are high-temperature phases and their energies are higher than for monoclinic HfO_2_ by more than 50 meV/atom^23^. In addition, c-BeO which was suggested to be a promising high-*k* candidate in ref.^20^ is also a metastable phase with the energy of 483 meV/atom with respect to the stable wurtzite BeO. Therefore, a more extensive table of *E*_g_-*k* relations for non-oxide compounds is in demand. We note that the open material database such as Materials Project^24^ or AFLOW^25^ provides *E*_g_ computed by the semilocal functional. However, the semilocal functional severely underestimates *E*_g_ such that it is not appropriate in screening dielectric materials, which requires accurate band gaps. In addition, these open databases do not provide dielectric constants. (Materials Project seems to open dielectric constants only for materials studied in ref.^21^.).

In this study, by conducting high-throughput *ab initio* calculations on accurate *E*_g_ and *k*, we screen high-*k* non-oxide materials such as carbides, nitrides, sulfides, phosphides, chloride and fluorides. Since the number of these compounds amounts to ~30,000 entries in the current the Inorganic Crystal Structure Database (ICSD)^26,27^, the computation on the whole materials is not feasible within our computational resource. Therefore, we first limit the screening to binary phases (869 structures) and compare *E*_g_-*k* relations depending on the anion species. We find that the *E*_g_-*k* relation of binary fluorides looks most promising and so extend the screening space to ternary fluorides (415 structures). Consequently, we identify candidate fluorides that are suitable for high-*k* dielectrics.

## Results

### Automation workflow

**Figure 1:**
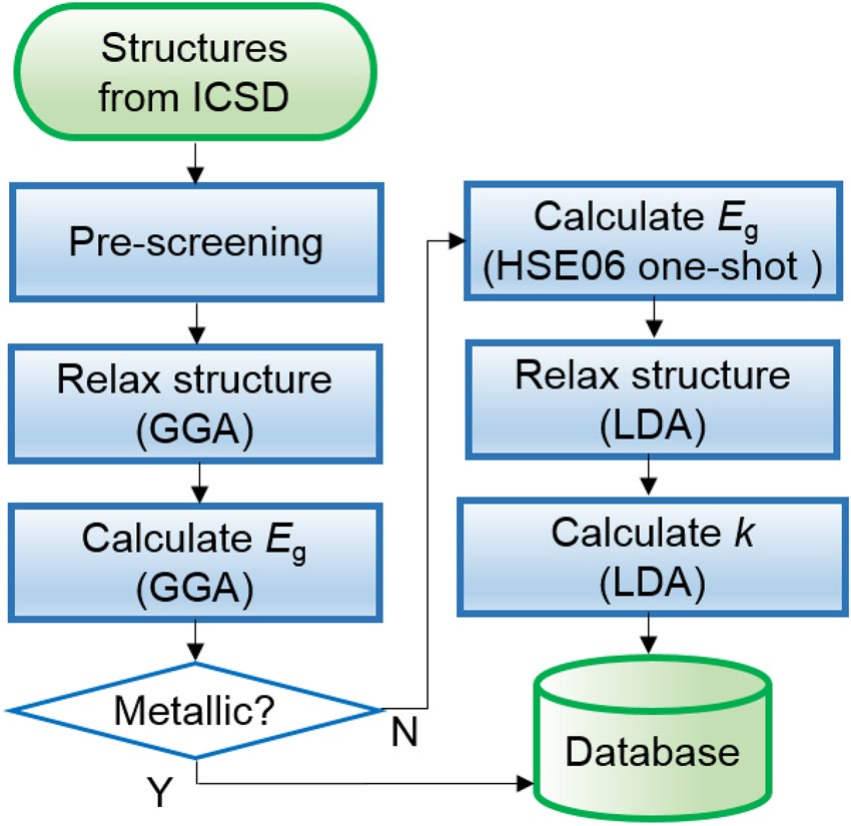
The workflow of calculating *E*_g_ and *k* for non-oxide compounds.

Figure 1 shows the overall workflow of the automated computations. First, from ICSD (the 2015 version), we garner structural information on ordered binary crystals that were experimentally identified and contain only one of carbon, nitrogen, fluorine, phosphorus, sulfur or chlorine atoms (non-oxide groups hereafter). We exclude compounds including 3*d* transition metal elements with partially occupied *d* orbitals (V ~ Cu) because their band gaps are usually smaller than 3 eV, and so they are not suitable for high-*k* applications. In addition, DFT + *U* methods that are necessary for 3*d* orbitals can significantly underestimate dielectric constants by hardening phonon modes^28^. We also omit large primitive cells that contain more than 50 atoms in the unit cell due to a sheer computational cost. (The total number of such structures is 53, and their dielectric constants are typically small.) After these pre-screening steps, we perform the structural relaxation and calculate *E*_g_ and *k* using the in-house automation package (Automated *Ab initio* Modeling of Materials Property Package (AMP^2^))^29^ for high-throughput calculations. (See the Methods section for further computational details.).

### Validation of automatic calculations

**Figure 2:**
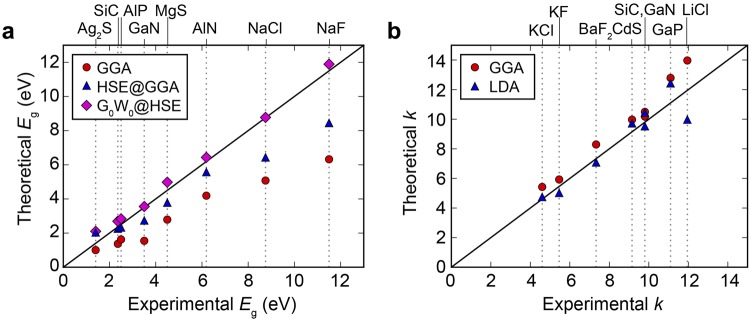
Comparison of experimental^57–63^ and theoretical data for (**a**) the band gap (*E*_g_) and (**b**) the static dielectric constant (*k*). HSE@GGA means a scheme applying the hybrid-functional calculations on the band-edge points identified by GGA. For the comparison purpose, results with G_0_W_0_@HSE are also presented.

In the present work, the band gap is calculated within the hybrid functional (HSE06) with structural parameters (lattice vectors and atomic coordinates) fixed to those obtained using the generalized gradient approximation (GGA) functional. To reduce the computational cost, we employ the HSE@GGA scheme in which the HSE calculation is performed on the band edge points identified by GGA. In ref.^20^, the HSE@GGA scheme was validated by comparing with experimental and other theoretical band gaps for selected oxides. Similarly, Fig. 2(a) tests this approach against various non-oxide compounds. The estimated band gaps are in good agreement with the experiment except for compounds with *E*_g_ > 8 eV that show noticeable discrepancies^30^. This is caused by the fixed fraction of the exact exchange term; it is known that materials with large *E*_g_ require higher fractions of the Fock term due to the weak electronic screening^31^. For a comparison purpose, we also present in Fig. 2(a) results with G_0_W_0_@HSE in which one-shot GW calculation is performed based on the HSE result^32^. It is seen that *E*_g_’s from G_0_W_0_@HSE are in very good agreement with experimental values. However, the GW method is too expensive to be used in the high-throughput screening.

For dielectric constants, we compare GGA and LDA results of selected compounds with experimental values (see Fig. 2(b)). The estimated dielectric constants are in good agreements with experiment regardless of the functional, though results show that GGA tends to give higher dielectric constant compared to LDA. The mean average deviation (MAD) is slightly lower with LDA than GGA (0.70 and 0.98, respectively). Considering that GGA tends to overestimate dielectric constants for high-*k* materials^20^, we employ the LDA scheme in evaluating dielectric constants. To note, we do not consider the HSE functional for evaluating *k* because HSE tends to underestimate the optical dielectric constant^32^. Furthermore, HSE is not superior to LDA for the static dielectric constant, as was demonstrated for TiO_2_^28^.

### High-throughput screening for binary non-oxides

**Figure 3:**
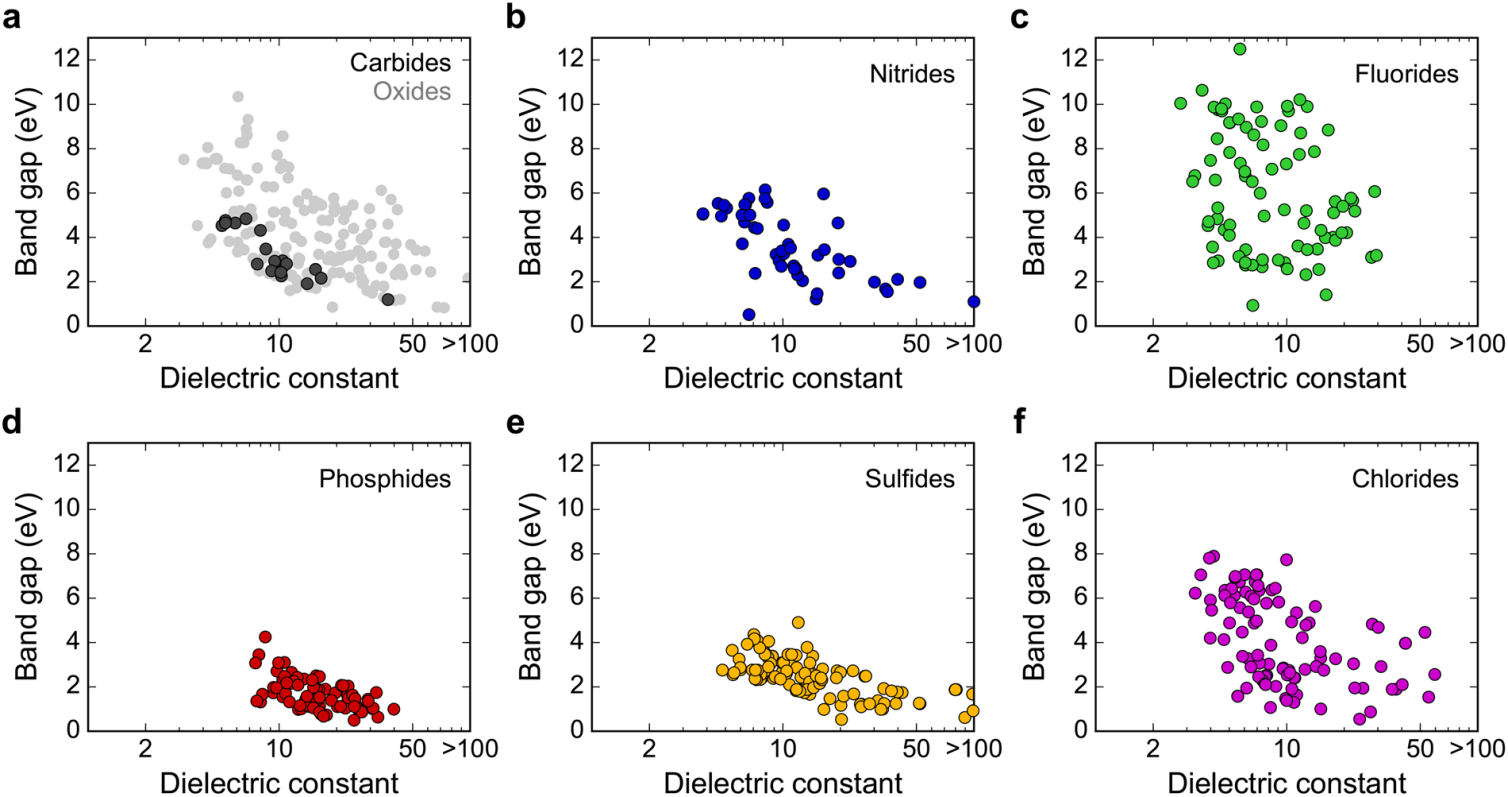
Band gap versus dielectric constants for (**a**) carbides and oxides^20^ (grey), (**b**) nitrides, (**c**) fluorides, (**d**) phosphides, (**e**) sulfides, and (**f**) chlorides. The number of data points are 20 (carbides), 49 (nitrides), 86 (fluorides), 82 (phosphides), 114 (sulfides), and 98 (chlorides).

For binary dielectrics, we consider 869 compounds (76 carbides, 123 nitrides, 132 fluorides, 205 sulfides, 194 phosphides and 139 chlorides). Among them, 20 carbides, 49 nitrides, 86 fluorides, 82 phosphides, 114 sulfides, and 98 chlorides are found to be insulators. The *E*_g_-*k* relation of each non-oxide group is presented in Fig. 3. All the numerical data are provided in Tables S1–S6 in Supplementary Information. For the comparison purpose, *E*_g_-*k* of oxides from ref.^20^ are also plotted as gray points in Fig. 3(a). The metallic compounds are not displayed since their dielectric constants are ill defined. We also exclude unstable structures that show imaginary optical modes in the unit cell calculation.

In Fig. 3, it is seen that the inverse relationship between *E*_g_ and *k* persist for every compound. However, the detailed distributions are distinct between the material groups. Especially, it is noticeable that fluorides have wide band gaps than other non-oxide groups. The band gap is related to the energy splitting between bonding and anti-bonding orbitals. Therefore, a large difference in electronegativity, small ionic size, and a high degree of orbital overlap contribute to strong bond strength and large band gap. Fluorine has the highest electronegativity in the Periodic Table and its ionic size is the smallest among considered anions. This results in a much broader *E*_g_ range than those of other non-oxides. The maximum band gap follows the order of fluorides > oxides > chlorides > nitrides > sulfides > carbides > phosphides, which is also in line with the order of electronegativity. The correlation between *E*_g_ and electronegativity was also discussed previously^33,34^.

### Distribution of dielectric constants

**Figure 4:**
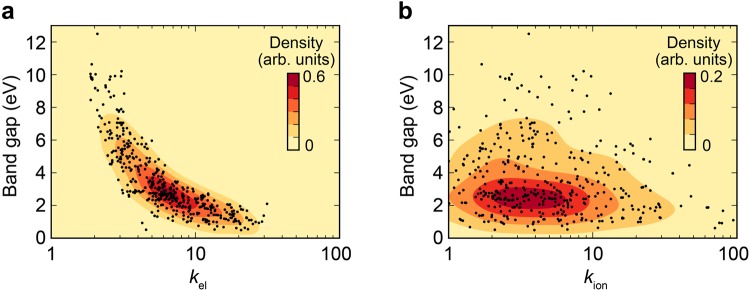
Dielectric constant versus band gap relation for binary non-oxides. The dielectric constant is split into the (**a**) electronic part (*k*_el_) and (**b**) ionic part (*k*_ion_). The black dots indicate each material. The density distribution of data points is visualized in contour plots.

In Fig. 3, the inverse relationship between *E*_g_ and *k* consistently appear, which puts a fundamental limitation on the existence of ideal high-*k* dielectrics that have large *E*_g_ and *k* simultaneously. For detailed analysis on this, we divide the dielectric constant into electronic and ionic contributions (*k*_el_ and *k*_ion_, respectively), and plot *E*_g_-*k*_el_ and *E*_g_-*k*_ion_ relations separately for all the data on binary compounds (see Figs. 4(a) and 4(b), respectively.) The density of data points is drawn in contours by representing each point with a Gaussian. In Fig. 4(a), a clear inverse relation is found between *E*_g_ and *k*_el_. This can be rationalized by the fact that the band gap reflects the bonding-antibonding separation and so a small *E*_g_ means higher electronic polarizability associated with the facile excitation into antibonding states, leading to higher *k*_el_. On the other hand, it is seen in Fig. 4(b) that the data points in *E*_g_-*k*_ion_ are more scattered such that the inverse relationship between *E*_g_ and *k*_ion_ is weaker than for between *E*_g_ and *k*_el_. The ionic dielectric constant is dictated by off-centering of the cations with respect to the anions (and vice versa) under electric fields, the degree of which depends on the bond strength. Compared to the band gap, the bond strength or phonon frequency is highly sensitive to the bond length. Therefore, various bond lengths among similar compositions result in a wide variation of *k*_ion_. Since the dielectric constant of high-*k* materials is mostly contributed by *k*_ion_, this weak correlation between *E*_g_ and *k*_ion_ increases a chance of finding new high-*k* materials by expanding the search space.

**Figure 5:**
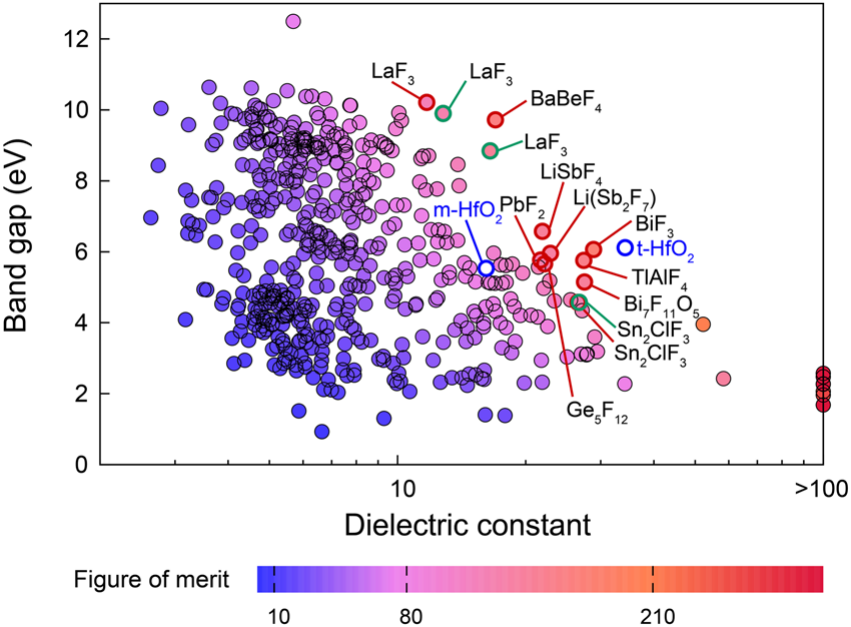
*E*_g_-*k* map for 86 binary and 415 ternary fluorides. Each material is color coded according to the figure of merit that is the product of *E*_g_ and *k*. The candidate fluorides for high-*k* dielectrics are marked in red (stable phase) or green (metastable phase) circles. As a reference, monoclinic and tetragonal HfO_2_ (m-HfO_2_ and t-HfO_2_, respectively) are also noted.

### High-throughput screening for ternary fluorides

Figure 3 indicates that band gaps of carbides, nitrides, phosphides and sulfides are distributed mostly over medium to small values and therefore, these material groups might not be appropriate for high-*k* applications. Both fluorides and chlorides follow similar *E*_g_-*k* relations but fluorides show a broader distribution. Considering the above discussion on a loose relation between *E*_g_ and *k*_ion_, it might be worthwhile to extend the search space to ternary fluorides. There are 644 ternary fluorides reported in ICSD and the calculated *E*_g_-*k* relations for 415 ternary fluorides with finite gaps are provided in Fig. 5, together with the binary fluorides presented in Fig. 3(c). The numerical data are compiled in Table S7 in Supplementary Information. To rank the candidate materials, we assign *E*_g_·*k* as the figure of merit (FOM) because *E*_g_ and *k* are approximately proportional to the logarithm of the leakage current density^20,35,36^. Data points in Fig. 5 are colored according to FOM.

In Fig. 5, we identify candidate fluorides that merit consideration as high-*k* dielectrics. For the candidate materials, we confirm the dynamical stability with phonon analysis and neglect dynamically unstable structures. (See the Methods section for details on the phonon calculations.) As a reference, monoclinic and tetragonal HfO_2_ (m-HfO_2_ and t-HfO_2_, respectively) are also marked. It is seen that no fluorides outperform tetragonal HfO_2_ (t-HfO_2_) that is currently industry-standard high-*k* dielectrics. However, t-HfO_2_ is a high-temperature phase and hence its stabilization at the room temperature requires strain engineering or dopants during device fabrication^37,38^. In contrast, many candidate materials in Fig. 5 are stable phases (9 stable and 3 metstable) and so synthesis would be more straightforward than HfO_2_.

## Discussions

**Figure 6:**
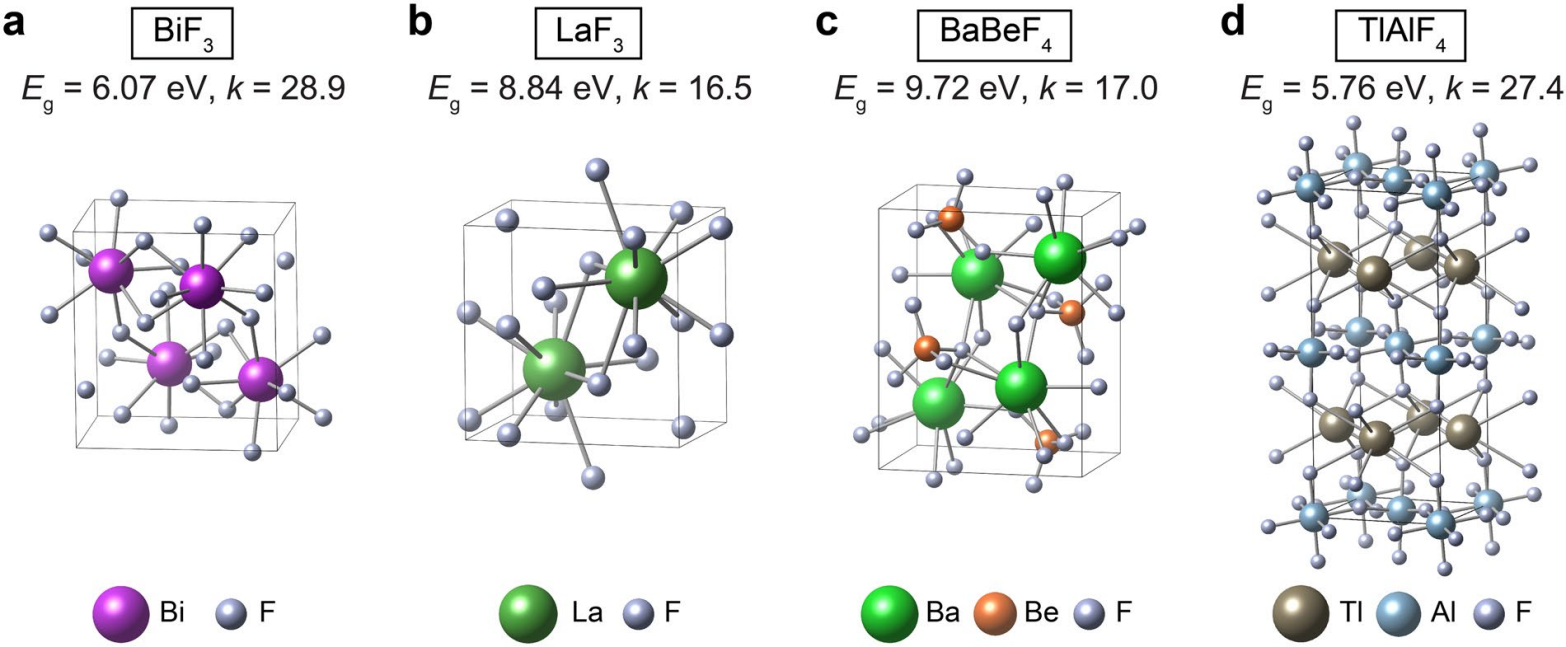
Crystal structures of (**a**) BiF_3_, (**b**) LaF_3_, (**c**) BaBeF_4_, and (**d**) TlAlF_4_.

In Table 1, we enlist candidate fluorides that were identified in Fig. 5. Besides *E*_g_ and *k* values, relative energies with respect to the most stable phase at ambient conditions are also provided. Some materials in Table 1 were also noted in ref.^21^ but the dielectric constants were larger than the present results because of the functional difference (see above). Among the binary phases, BiF_3_ (see Fig. 6(a)) looks promising with *E*_g_ and *k* values close to those of t-HfO_2_. BiF_3_ was used as dielectric buffer layer for surface plasmon resonance^39^. However, unlike t-HfO_2_ that is metastable, BiF_3_ is the stable phase and therefore we expect that the fabrication would be easier, and the film quality would be more uniform than HfO_2_. The polymorphs of LaF_3_ are also intriguing as they possess large band gaps of 9~10 eV. LaF_3_ was used as dielectric buffer layer^39^ or UV coating^40^. There are several polymorphs in LaF_3_ and their dielectric constants range over 11~17 with metastable phases showing larger values (see Table S3). The *Pmmn* structure with the largest *k* is shown in Fig. 6(b).

**Table 1:** Candidate fluorides for high-*k* dielectrics with *E*_g_ > 4 eV and figure of merit (FOM) > 120. ∆*E* means the energy difference with respect to that of the stable phase.

Name	ICSD number	Space group	*E*_g_ (eV)	*k*	FOM	∆*E* (eV/atom)
BiF_3_	9015	*Pnma*	6.07	28.9	175.2	0.0
BaBeF_4_	414412	*Pnma*	9.72	17.0	165.0	0.0
Tl(AlF_4_)	202458	*C*2/*c*	5.76	27.4	157.6	0.0
LaF_3_	167553	*Pmmn*	8.84	16.5	145.9	0.202
LiSbF_4_	428177	*P*2_1_3	6.58	21.9	144.1	0.0
Bi_7_F_11_O_5_	167074	*C*2	5.15	27.5	141.5	0.0
Li(Sb_2_F_7_)	428176	*Pnma*	5.96	22.9	136.2	0.0
LaF_3_	34108	*P*6_3_/*mmc*	9.90	12.8	126.6	0.014
Ge_5_F_12_	10295	*P*2_1_/*c*	5.66	22.1	125.3	0.0
PbF_2_	76420	*Fm*-3*m*	5.77	21.7	124.9	0.0
Sn_2_ClF_3_	2088	*P*2_1_3	4.57	26.7	122.2	0.0

Fluorides are sorted in the decreasing order of FOM.

There are several Ge-F compounds such as Ge_3_F_8_, Ge_5_F_12_, GeF_2_, and GeF_4_. The band gap of these compounds range over 5~7.5 eV. (See Table S3) This is in contrast with GeO_2_ whose band gap is only 2.8 eV. The small band gaps of GeO_2_ and suboxides result in high-density defect states, degrading the interface quality in Ge transistors^41^. In this respect, the large band gap of germanium fluorides may contribute to forming stable interfaces in Ge devices by playing as the passivation layer that removes interface defects.

Among the ternary fluorides, BaBeF_4_ is noticeable as the material possess a large band gap of 9.7 eV and dielectric constant of 17, surpassing that of LaF_3_. (See Fig. 6(c).) We note that tetragonal TlAlF_4_, which has the highest FOM in ref.^21^, is found to be dynamically unstable in the present calculation (both LDA and GGA) while dynamically stable monoclinic TlAlF_4_ (see Fig. 6(d)) has *k* of 27. Nevertheless, the dielectric property of monoclinic TlAlF_4_ approaches that of t-HfO_2_.

In summary, we conducted high-throughput calculations of *E*_g_ and *k* for 449 binary non-oxides and 415 ternary fluorides. We confirmed that inverse relationships between *E*_g_ and *k* are present in non-oxide compounds like in oxide compounds. Among the different anion groups, binary fluorides are the most promising as they show a wide distribution of *E*_g_. By further screening over ternary fluorides, we identified fluorides such as BiF_3_, LaF_3_, and BaBeF_4_ that could serve as useful high-*k* dielectrics. We believe that the suggested fluoride compounds may contribute to resolving various issues in microelectronic devices that is caused by using only oxide dielectrics.

## Methods

### Computational details

The DFT calculations were performed using Vienna *ab initio* simulation package (VASP)^42–44^ based on the projector augmented wave (PAW) pseudopotential^45,46^. For the exchange-correlation functional, we employ the generalized gradient approximation (GGA) in the form of the Perdew-Burke-Ernzerhof (PBE)^47^. Compounds bearing Zn, La, and Ce are performed with the GGA + *U* method^48^. The effective *U* parameter of 7.5 eV is used for Zn *d* and La *f* orbitals^49,50^ while 4.5 eV is used for Ce *f* orbitals^51^. We also carry out the hybrid functional (HSE06) calculation^52^ to overcome underestimation of the band gap in GGA. The **k**-point meshes are selected by ensuring that the total energy, stress tensor components, and all forces are converged within 10 meV/atom, 10 kbar, and 0.02 eV Å^−1^, respectively. The atomic positions and lattice parameters are relaxed until the total energy, atomic forces, and stress tensors are reduced to within the same criteria.

### Band gap

We adopt an automated approach in estimating the band gap as was detailed in our former high-throughput study^20^. To be brief, we first identify **k** points corresponding to the valence band maximum and conduction band minimum by sweeping along the lines connecting high-symmetry points^53^ using GGA(+*U*) functional. Then, we perform the one-shot hybrid functional calculation on these band-edge points.

### Dielectric constant

The density-functional perturbation theory (DFPT)^54,55^ implemented in VASP is used to estimate Born effective charges, phonon frequencies, and dielectric constants. In calculating the dielectric constants, the **k**-point density is doubled because DFPT is sensitive to this computational parameter.

### Phonon analysis

Phonon bands are calculated using the PHONOPY package^56^.

## Electronic supplementary material

Supplementary information

## Data Availability

All data sets used in this work are available from the corresponding author on reasonable request.
