# Supplementary material for: High-throughput ab initio calculations on dielectric constant and band gap of non-oxide dielectrics
Source: Sci Rep. 2018 Oct 4;8:14794. doi: 10.1038/s41598-018-33095-6 (PMC6172237; doi:10.1038/s41598-018-33095-6)
Supplement: Supplementary file 1 — Supplementary information [file 41598_2018_33095_MOESM1_ESM.pdf]

High-throughput *ab initio* calculations on dielectric constants and band gap of non-oxide dielectrics

Miso Lee[1], Yong Youn[1], Kanghoon Yim[2]* & Seungwu Han[1]*

[1]Department of Materials Science and Engineering and Research Institute of Advanced Materials, Seoul National University, Seoul 08826, Korea

[2]Korea Institute of Energy Research, Daejeon 34129, Korea

Supplementary information

Table S1. Names, ICSD numbers, space groups, band gaps, dielectric tensors, and ΔE of binary carbides.

Name	ICSD	Space group	E_g (eV)	Dielectric tensor	ΔE (meV/atom)
$(B_{12}C)C_2$	612562	166	2.49	8.3, 8.3, 10.7	0
Al_4C_3	14397	160	2.16	15.8, 15.8, 18.0	0
Al_4C_3	654940	166	2.16	15.0, 15.0, 18.1	0.01
BaC_2	88101	139	2.81	9.0, 11.5, 11.5	0
BaC_2	186576	166	2.56	15.1, 15.1, 16.1	80.68
Be_2C	616185	225	1.91	14.0, 14.0, 14.0	0
CSe_2	60374	64	2.79	5.3, 7.3, 10.3	0
Cs_2C_2	51534	62	4.31	7.4, 7.5, 8.9	0
K_2C_2	89529	142	4.53	4.8, 5.0, 5.0	0
Li_2C_2	89535	71	4.65	5.7, 5.8, 6.0	0
Mg_2C_3	71941	58	2.42	6.0, 9.5, 14.3	0
Na_2C_2	89527	142	4.65	5.0, 5.3, 5.3	0
Na_2C_2	95835	71	4.76	5.1, 5.2, 5.3	1.8
Rb_2C_2	51532	62	4.84	6.0, 6.5, 7.2	0
SiC	603798	216	2.26	10.2, 10.2, 10.2	0
SiC	164429	186	2.94	10.2, 10.2, 10.7	7.85
SiC	107204	156	2.66	10.2, 10.2, 10.7	9.12
SrC_2	91051	15	3.47	8.0, 8.2, 8.8	0
SrC_2	91050	139	2.93	7.0, 10.2, 10.2	11.43
Y_4C_7	658826	14	1.19	29.3, 41.0, 41.1	0

Table S2. Names, ICSD numbers, space groups, band gaps, dielectric tensors, and ΔE of binary nitrides.

Name	ICSD	Space group	E_g (eV)	Dielectric tensor	ΔE (meV/atom)
AgN_3	183201	140	2.71	11.0, 11.5, 11.5	0
AlN	183638	186	5.58	7.7, 7.7, 9.2	0
AlN	105522	225	5.96	16.3, 16.3, 16.3	159.77
$As(N_3)_3$	413360	14	3.68	9.0, 9.5, 12.5	0
BN	240996	187	5.48	4.6, 7.1, 7.1	0
BN	24644	194	5.47	4.8, 7.1, 7.1	1.21
BN	77374	186	4.69	3.8, 7.5, 7.5	2.72
BN	27879	216	5.76	6.6, 6.6, 6.6	52.65
BN	20946	42	3.71	4.0, 7.1, 7.1	84.44
BN	27986	164	0.51	6.6, 6.6, 6.6	2035.87
$Ba(N_3)_2$	26202	11	5	4.5, 6.5, 7.1	0
$Ba(N_3)_2$	412253	11	5.01	4.5, 6.5, 7.2	1.63
Be_3N_2	616348	206	4.56	10.0, 10.0, 10.0	0
Be_3N_2	25656	194	4.65	10.8, 10.8, 36.5	30.63
BrN_3	423741	110	3.23	6.0, 10.5, 10.5	0
Ca_3N_2	169725	206	2.04	12.6, 12.6, 12.6	0
Ca_3N_2	169726	12	2.39	17.0, 18.8, 22.6	50.68
Ca_3N_2	169727	164	2.91	20.4, 20.6, 26.3	102.51
CsN_3	627047	140	5.32	4.5, 5.3, 5.3	0
GaN	157398	186	2.94	9.2, 9.2, 10.1	0
GaN	41500	225	1.67	34.4, 34.4, 34.4	404.25
Ge_3N_4	637162	159	3.39	9.8, 9.8, 9.8	0
Ge_3N_4	23672	176	3.26	10.0, 10.0, 10.1	0.41
Ge_3N_4	87767	227	3.2	15.2, 15.2, 15.2	61.43
Hf_3N_4	97997	220	1.98	30.1, 30.1, 30.1	0
$Hg(N_3)_2$	426343	45	3.52	6.0, 12.7, 13.6	0
KN_3	34269	140	5.53	3.6, 5.0, 5.0	0
Li_3N	156889	194	2.31	11.0, 11.0, 13.1	0
LiN_3	34675	12	5.01	4.7, 5.3, 10.1	0
Mg_3N_2	84917	206	2.69	9.8, 9.8, 9.8	0
N_4Se_4	14325	15	2.58	10.8, 11.6, 11.9	0

Name	ICSD	Space group	E_g (eV)	Dielectric tensor	ΔE (meV/atom)
NaN_3	34267	166	5.06	2.9, 2.9, 5.5	0
NaN_3	644523	160	5.04	3.0, 3.2, 5.1	2.2
RbN_3	34272	140	5.45	3.9, 5.4, 5.4	0
RbN_3	16963	123	4.96	3.8, 3.8, 6.0	19.43
$Sb(N_3)_3$	422405	2	3.44	12.4, 17.6, 19.3	0
$Sb(N_3)_3$	413359	148	3.01	12.9, 22.0, 23.8	7.74
Se_4N_4	74838	14	2.58	10.0, 12.1, 12.4	0
Si_3N_4	170003	173	5.75	8.0, 8.0, 8.0	0
Si_3N_4	164618	159	6.14	7.9, 8.1, 8.1	2.36
Si_3N_4	67241	173	4.44	6.8, 6.8, 7.5	230.85
Sn_3N_4	89525	227	1.46	15.0, 15.0, 15.0	0
Ta_3N_5	66533	63	2.1	34.8, 36.5, 48.1	0
$W(N_3)_6$	413860	147	2.38	6.9, 6.9, 7.5	0
YN	76528	225	1.1	111.2, 111.2, 111.2	0
$Zn(N_3)_2$	430430	5	4.4	6.9, 7.0, 7.5	0
Zn_3N_2	84918	206	1.22	14.9, 14.9, 14.9	0
Zr_3N_4	78944	62	1.97	46.3, 50.0, 59.7	0
Zr_3N_4	97998	220	1.55	35.2, 35.2, 35.2	42.68

Table S3. Names, ICSD numbers, space groups, band gaps, dielectric tensors, and ΔE of binary fluorides

Name	ICSD	Space group	E_g (eV)	Dielectric tensor	ΔE (meV/atom)
$(SbF_3)_3(SbF_5)$	35709	11	5.11	7.0, 14.4, 31.2	0
$(SnF)_2(SnF_6)$	32592	14	4.32	8.4, 14.5, 22.2	0
$(XeF_2)(XeF_4)$	18128	14	4.09	4.6, 5.0, 5.3	0
AlF_3	29131	150	9.8	4.5, 4.5, 4.5	0
AlF_3	130021	221	9.7	4.5, 4.5, 4.5	0.23
AlF_3	202681	63	9.87	4.0, 4.1, 4.3	0.55
AlF_3	30274	155	10.02	4.7, 4.7, 4.8	1.95
AlF_3	79816	127	9.75	4.3, 4.3, 4.4	3.46
AsF_3	35132	33	6.77	5.4, 5.5, 7.0	0

Name	ICSD	Space group	E_g (eV)	Dielectric tensor	ΔE (meV/atom)
$Au(AuF_4)_2$	89620	14	2.67	5.2, 7.1, 9.9	0
AuF_5	411877	62	2.75	4.8, 5.0, 8.3	0
$B_{10}F_{12}$	412618	88	4.53	3.7, 3.7, 4.1	0
B_2F_4	27867	14	6.78	3.0, 3.3, 3.5	0
BaF_2	41649	225	8.97	6.1, 6.1, 6.1	0
BaF_2	41651	62	7.74	10.3, 10.3, 14.2	146.77
BeF_2	261194	152	10.63	3.5, 3.5, 3.6	0
BiF_3	9015	62	6.07	24.6, 28.9, 32.9	0
BiF_5	25023	87	3.47	8.8, 8.8, 25.6	0
CaF_2	60371	225	9.23	7.3, 7.3, 7.3	0
CaF_2	656449	62	9.7	9.7, 9.9, 10.9	55
CdF_2	183500	225	4.96	7.6, 7.6, 7.6	0
CeF_3	81674	165	4.64	12.1, 12.2, 12.5	0
CsF	53832	225	7.08	8.3, 8.3, 8.3	0
CsF	61563	221	7.86	13.9, 13.9, 13.9	108.82
GaF_3	409507	167	6.97	5.7, 5.7, 6.6	0
Ge_3F_8	427896	14	5.4	9.3, 16.0, 33.4	0
Ge_5F_{12}	10295	14	5.66	19.7, 23.1, 23.5	0
GeF_2	18030	19	5.18	15.7, 24.4, 28.0	0
GeF_4	202558	217	7.47	3.9, 3.9, 3.9	0
Hg_2F_2	27700	139	2.55	11.0, 16.0, 16.0	0
HgF_2	33614	225	2.86	9.7, 9.7, 9.7	0
IF_3	411036	62	3.61	4.1, 7.7, 22.0	0
InF_3	38306	167	5.99	7.1, 7.1, 7.4	0
IrF_3	77619	167	2.99	7.2, 7.2, 7.9	0
IrF_6	171654	62	2.85	4.0, 4.1, 4.1	0
KF	64686	225	7.83	5.0, 5.0, 5.0	0
KF	61558	221	8.17	7.5, 7.5, 7.5	102.64
KrF_2	23534	136	4.71	2.5, 4.5, 4.5	0
KrF_2	279623	139	4.84	2.8, 2.8, 7.2	7
LaF_3	246323	185	10.21	10.7, 12.2, 12.2	0
LaF_3	34108	194	9.9	11.7, 11.7, 14.9	14.08

Name	ICSD	Space group	E_g (eV)	Dielectric tensor	ΔE (meV/atom)
LaF_3	167553	59	8.84	15.4, 15.5, 18.0	201.8
LiF	41409	225	12.5	5.6, 5.6, 5.6	0
MgF_2	8120	136	9.18	4.3, 5.3, 5.3	0
MgF_2	422263	58	9.17	4.3, 5.3, 5.4	0.18
MgF_2	94282	205	9.04	9.3, 9.3, 9.3	66.96
MoF_3	68527	167	3.44	5.7, 5.7, 6.7	0
MoF_3	30612	221	2.59	137.8, 137.8, 137.8	45.76
MoF_6	36219	62	5.33	4.3, 4.3, 4.3	0
NaF	262837	225	8.45	4.3, 4.3, 4.3	0
Nb_6F_{15}	415950	229	0.93	6.6, 6.6, 6.6	0
OsF_5	27227	14	2.94	4.0, 4.4, 4.6	0
PbF_2	76420	225	5.77	21.6, 21.6, 21.6	0
PbF_4	78895	139	3.45	6.0, 16.0, 16.0	0
PdF_4	1555	43	2.75	4.8, 7.0, 7.2	0
PtF_4	71579	43	3.14	4.2, 6.1, 6.4	0
RbF	53828	225	7.34	5.7, 5.7, 5.7	0
RbF	61562	221	8.62	6.7, 6.7, 6.7	124.67
ReF_7	78311	2	3.56	3.8, 4.0, 4.3	0
RhF_3	62262	167	2.98	8.6, 8.6, 9.7	0
RhF_3	29134	150	1.41	15.0, 15.0, 18.0	95.35
RuF_4	165398	14	2.58	6.7, 11.2, 12.2	0
RuF_5	27226	14	2.86	5.6, 6.1, 6.3	0
SbF_3	16142	40	5.61	13.8, 14.7, 25.2	0
SiF_4	24500	217	10.04	2.7, 2.7, 2.7	0
SnF_2	14195	92	4.21	17.7, 17.7, 22.8	0
SnF_2	14194	19	4.21	16.4, 22.0, 23.2	0.45
SnF_2	308	15	3.19	19.4, 25.1, 43.7	82.8
SnF_4	78894	139	5.24	4.7, 12.2, 12.2	0
SrF_2	40414	225	9.34	5.5, 5.5, 5.5	0
SrF_2	262349	62	9.88	6.7, 6.8, 7.2	60.02
SrF_2	262350	194	8.71	9.2, 9.2, 17.0	190.34
TeF_4	85452	19	5.2	5.9, 12.4, 19.6	0

Name	ICSD	Space group	E_g (eV)	Dielectric tensor	ΔE (meV/atom)
TeF_6	67609	62	6.52	3.2, 3.2, 3.2	0
TlF	90994	57	3.87	10.6, 21.5, 21.7	0
TlF	90993	129	3.99	9.0, 19.3, 19.3	6.29
TlF	9873	28	4	10.7, 20.7, 20.8	8.67
TlF	9893	139	3.1	25.9, 28.6, 28.6	38.03
TlF	30268	69	3.12	27.4, 27.6, 28.5	42.72
TlF_3	18029	62	2.32	8.9, 11.1, 17.0	0
WF_6	81854	62	6.59	4.2, 4.2, 4.2	0
XeF_2	260950	139	4.34	3.7, 3.7, 6.5	0
XeF_4	27467	14	4.56	4.2, 5.4, 5.4	0
YF_3	26595	62	9.92	8.8, 9.0, 12.3	0
ZnF_2	20364	60	6.51	6.0, 6.0, 7.0	0
ZrF_4	35100	84	7.31	9.7, 10.0, 10.0	0

Table S4. Names, ICSD numbers, space groups, band gaps, dielectric tensors, and ΔE of binary phosphides.

Name	ICSD	Space group	E_g (eV)	Dielectric tensor	ΔE (meV/atom)
Ag_3P_{11}	26563	8	1.43	13.0, 13.2, 14.2	0
AlP	24490	216	2.33	10.4, 10.4, 10.4	0
$B_{12}P_2$	62748	166	3.44	7.0, 7.0, 8.0	0
B_6P	615157	166	3.45	7.0, 7.0, 8.0	0
BP	615154	216	1.97	9.3, 9.3, 9.3	0
BP	615155	186	1.73	9.1, 9.1, 9.6	12.48
Ba_3P_{14}	653664	14	2.42	13.2, 14.4, 18.6	0
Ba_3P_{14}	23629	14	2.42	13.2, 13.4, 19.5	0.09
Ba_3P_4	38322	43	1.1	16.9, 20.0, 23.5	0
BaP_{10}	35295	36	1.89	13.2, 16.9, 17.2	0
BaP_3	23618	12	1.15	15.0, 15.4, 18.0	0
BaP_8	96544	2	1.42	14.7, 16.0, 17.0	0
Be_3P_2	616384	206	1.47	15.6, 15.6, 15.6	0
BeP_2	2262	1	1.55	10.2, 10.5, 10.5	0
Ca_2P_2	83352	189	1.01	10.6, 10.6, 17.7	0

Name	ICSD	Space group	E_g (eV)	Dielectric tensor	ΔE (meV/atom)
Ca_5P_8	74854	12	1.89	15.7, 17.0, 19.6	0
CaP	26261	189	0.98	10.4, 10.4, 16.8	0
CaP_3	74479	2	0.49	20.8, 24.0, 28.8	0
Cd_7P_{10}	200596	43	1.73	18.6, 20.1, 20.4	0
CdP_2	620214	33	2.16	10.9, 11.0, 12.7	0
CdP_2	620210	92	2.17	11.3, 11.8, 11.8	4.12
CdP_2	16500	96	2.17	11.5, 11.6, 11.6	5.02
CdP_4	620212	14	0.99	20.3, 35.5, 63.7	0
Cs_3P_7	62259	76	3.08	7.2, 7.6, 7.6	0
CsP_7	428141	57	1.96	8.0, 8.9, 12.0	0
GaP	77087	216	2.43	12.4, 12.4, 12.4	0
GeP	637492	12	1.11	24.1, 24.5, 28.0	0
InP	53105	216	1.12	14.2, 14.2, 14.2	0
IrP_2	174229	14	1.17	21.6, 21.9, 22.4	0
IrP_3	640899	204	0.73	17.7, 17.7, 17.7	0
K_3P	25550	194	0.75	12.9, 12.9, 23.9	0
K_4P_6	33260	70	1.67	6.1, 8.9, 9.4	0
K_4P_6	33259	69	1.32	6.1, 8.0, 8.9	0.53
KP	14010	19	1.7	8.0, 10.1, 13.5	0
LaP_2	42015	9	1	24.4, 25.1, 28.0	0
Li_3P	240861	194	1.32	11.1, 11.1, 11.7	0
Li_3P_7	60774	19	2.47	10.0, 10.5, 10.8	0
LiP	642222	14	1.39	16.6, 19.5, 20.0	0
LiP_5	88710	33	1.88	14.2, 14.6, 18.0	0
LiP_5	23620	33	1.88	14.0, 14.7, 18.8	0.05
Mg_3P_2	642724	206	2.25	11.9, 11.9, 11.9	0
MgP_4	42030	14	1.18	12.5, 14.1, 15.0	0
Na_3P	171012	194	1.05	13.5, 15.0, 15.0	0
NaP	14009	19	1.57	11.7, 13.4, 16.7	0
OsP_2	238252	58	1.44	28.0, 28.2, 31.3	0
OsP_4	647710	2	2.07	19.6, 21.8, 22.0	0
P_2I_4	203216	2	2.71	7.7, 9.9, 11.5	0

Name	ICSD	Space group	E_g (eV)	Dielectric tensor	ΔE (meV/atom)
P_4Se_4	74878	14	3.08	8.0, 8.8, 12.8	0
P_4Se_5	16140	33	2.31	13.6, 13.7, 17.3	0
PBr_3	8052	62	4.25	4.9, 6.0, 14.0	0
PBr_5	15559	57	2.65	9.4, 12.2, 13.1	0
PBr_7	26025	62	2.35	9.4, 13.5, 17.5	0
PI_3	311	173	3.1	9.1, 11.4, 11.4	0
PbP_7	427804	14	1.36	25.0, 25.9, 36.8	0
PdP_2	48163	15	1.01	24.6, 29.4, 38.5	0
PtP_2	71029	205	1.61	22.7, 22.7, 22.7	0
Rb_2P_3	654296	69	1.36	6.2, 8.2, 8.4	0
Rb_4P_6	65184	69	1.36	6.2, 8.2, 8.4	0
Re_2P_5	24808	2	1.11	23.9, 24.5, 25.8	0
ReP_3	647985	62	0.63	29.7, 33.9, 34.9	0
ReP_4	8197	61	1.47	22.8, 24.5, 25.6	0
RhP_2	174223	14	0.86	26.7, 27.0, 27.5	0
RuP_2	42607	58	1.05	29.0, 29.5, 33.0	0
RuP_3	62420	2	2.03	22.2, 23.2, 23.4	0
RuP_4	2492	2	2.06	20.3, 22.0, 22.3	0
RuP_4	648018	14	1.5	21.3, 23.4, 24.3	19.33
SiP	23724	36	2.08	11.1, 13.0, 13.4	0
SiP_2	43098	55	1.98	14.4, 14.9, 16.0	0
Sr_3P_{14}	42461	14	2.46	14.4, 14.6, 19.6	0
Sr_3P_{14}	648173	14	2.5	13.9, 15.5, 17.3	6.96
Sr_3P_4	38321	43	1.52	14.0, 16.5, 18.2	0
SrP	26262	189	1.15	10.3, 10.3, 17.9	0
SrP_3	23628	12	0.86	14.8, 16.2, 17.8	0
TcP_3	35200	62	1.04	28.2, 31.0, 33.5	0
TcP_4	35117	61	1.62	22.9, 24.0, 25.4	0
TlP_5	15021	26	1.74	20.0, 20.5, 56.2	0
YP_5	409188	11	0.68	16.0, 16.2, 18.5	0
Zn_3P_2	603896	137	1.11	18.0, 22.4, 22.4	0
Zn_3P_2	648310	206	1.04	22.2, 22.2, 22.2	17.58

Name	ICSD	Space group	E_g (eV)	Dielectric tensor	ΔE (meV/atom)
ZnP_2	250014	96	2.18	10.5, 11.1, 11.1	0
ZnP_2	601257	92	2.19	10.5, 11.1, 11.1	0.03
ZnP_4	40428	92	1.46	19.3, 27.0, 27.0	0

Table S5. Names, ICSD numbers, space groups, band gaps, dielectric tensors, and Δ*E* of binary sulfides.

Name	ICSD	Space group	E_g (eV)	Dielectric tensor	ΔE (meV/atom)
Al_2S_3	609250	167	3.38	13.3, 13.3, 16.3	0
Al_2S_3	609251	141	2.61	10.0, 10.0, 10.3	9.48
As_2S_3	25792	14	2.66	10.8, 23.7, 25.3	0
As_2S_3	655775	14	2.72	11.1, 22.4, 24.0	5.35
As_4S_3	16145	62	2.86	10.2, 12.4, 12.7	0
As_4S_3	16105	62	3.02	10.5, 11.1, 11.9	2.8
As_4S_4	185032	14	2.41	11.3, 11.8, 14.5	0
As_4S_4	80125	14	2.59	13.0, 13.3, 14.4	20.87
As_4S_4	360	14	2.63	12.6, 14.0, 15.3	24.41
As_8S_9	194921	13	2.34	12.0, 12.4, 13.6	0
AsS	24661	14	2.36	11.7, 13.0, 15.0	0
B_8S_{16}	15268	14	3.65	3.8, 6.0, 6.3	0
Ba_2S_3	70058	109	2.61	10.2, 14.4, 14.4	0
BaS	616053	225	3.08	13.8, 13.8, 13.8	0
BaS_2	2004	15	2.67	8.4, 10.2, 11.0	0
BaS_3	70059	113	2.43	10.9, 13.1, 13.1	0
BaS_3	26765	18	1.94	9.8, 11.0, 17.0	23.32
BeS	44724	216	4.1	7.0, 7.0, 7.0	0
CaS	619529	225	3.42	11.7, 11.7, 11.7	0
CdS	192569	216	2.1	10.8, 10.8, 10.8	0
CdS	154186	186	2.38	8.7, 8.7, 9.1	48.62
CdS	600773	59	1.21	25.1, 25.1, 25.9	130.19
CdS	52825	225	1.22	25.7, 25.7, 25.7	131.52
CdS_2	620305	205	2.22	13.7, 13.7, 13.7	0
Cs_2S	183207	62	3.09	9.4, 9.7, 10.0	0

Name	ICSD	Space group	E_g (eV)	Dielectric tensor	ΔE (meV/atom)
Cs_2S	183208	62	3.09	9.4, 9.7, 10.4	0.06
Cs_2S_2	200474	71	2.77	5.1, 6.2, 6.5	0
Cs_2S_3	14094	36	2.41	6.4, 6.8, 10.0	0
Cs_2S_5	201073	19	2.76	6.5, 6.6, 7.5	0
Cs_2S_6	428612	2	2.57	6.6, 6.8, 10.0	0
Ga_2S_3	409550	9	2.72	9.2, 9.5, 12.6	0
Ga_2S_3	488	9	3.09	9.0, 9.1, 11.7	8.91
GaS	40824	166	2.46	6.8, 10.2, 10.2	0
GeS	1256	62	1.77	27.6, 41.3, 50.1	0
GeS	653896	62	1.74	27.9, 41.6, 56.6	0.34
GeS_2	1947	14	3.33	6.0, 8.9, 10.0	0
GeS_2	44	7	3.37	6.9, 9.2, 10.1	8.31
GeS_2	31685	43	3.38	6.9, 9.2, 10.1	9.65
GeS_2	167194	122	3.46	10.1, 10.1, 11.5	14.4
GeS_2	85527	142	3.31	8.4, 8.4, 9.2	15.5
HfS_2	601164	164	1.92	8.2, 46.4, 46.4	0
HfS_3	638846	11	1.86	7.3, 12.2, 17.2	0
HgS	81923	152	2.49	24.0, 24.4, 32.6	0
HgS	639165	154	2.48	23.8, 23.9, 32.7	0.39
K_2S	183837	225	3.25	5.8, 5.8, 5.8	0
K_2S_2	43406	189	2.55	4.7, 4.7, 6.0	0
K_2S_3	1263	36	2.33	5.6, 7.5, 9.6	0
K_2S_5	641320	19	2.71	7.6, 7.8, 8.5	0
K_2S_6	247958	14	2.55	6.4, 7.0, 9.4	0
Li_2S	642291	225	4.35	7.0, 7.0, 7.0	0
Li_2S	91284	62	4.9	10.5, 11.0, 13.0	54.84
Li_2S	91283	33	4.08	7.2, 7.3, 7.5	173.98
MgS	41234	225	3.78	13.3, 13.3, 13.3	0
MoS_2	644250	194	1.73	7.7, 15.5, 15.5	0
MoS_2	38401	160	1.66	8.8, 16.3, 16.3	0.04
Na_2S_2	644955	194	2.33	5.8, 5.8, 10.0	0
Na_2S_4	2586	122	3.06	8.2, 8.8, 8.8	0

Name	ICSD	Space group	E_g (eV)	Dielectric tensor	ΔE (meV/atom)
Na_2S_5	644956	62	2.75	5.0, 7.8, 8.3	0
NaS	644958	194	2.32	5.8, 5.8, 10.0	0
PbS	68712	5	0.62	82.1, 82.9, 103.7	0
PbS	62190	225	0.92	98.9, 98.9, 98.9	22.94
PbS	648438	62	0.93	94.7, 106.0, 108.4	26.43
PbS	250761	63	1.67	25.0, 112.6, 156.6	75.32
PtS	649539	84	1.17	19.9, 19.9, 20.4	0
PtS_2	649534	164	1.38	23.5, 35.3, 35.4	0
Rb_2S	29208	225	2.87	5.9, 5.9, 5.9	0
Rb_2S	261444	62	2.9	8.1, 8.1, 8.8	27.53
Rb_2S	261443	194	2.8	10.5, 10.5, 27.2	90.68
Rb_2S_2	73176	189	2.64	4.8, 4.8, 6.9	0
Rb_2S_2	73175	71	2.76	4.4, 4.8, 5.0	6.17
Rb_2S_3	14092	36	2.36	5.0, 6.8, 8.9	0
Rb_2S_5	100321	19	2.75	6.7, 7.0, 7.7	0
ReS_2	650077	2	1.88	7.0, 16.1, 16.4	0
ReS_2	81814	2	1.88	8.4, 15.9, 16.1	1.3
Rh_2S_3	15344	60	1.1	24.9, 25.6, 26.5	0
RuS_2	56019	205	1.58	20.7, 20.7, 20.7	0
Sb_2S_3	99799	62	1.87	20.8, 108.7, 110.3	0
Sb_2S_3	85302	31	1.88	21.0, 108.5, 113.2	0.18
Sb_2S_3	425648	62	1.88	20.9, 108.1, 110.7	0.93
Sb_2S_3	26751	62	1.87	20.8, 108.8, 109.0	0.99
Sb_2S_3	95558	62	1.88	20.9, 108.9, 111.0	1.08
SiS_2	26858	72	3.92	4.8, 5.2, 9.4	0
SiS_2	291212	14	4.17	5.5, 7.5, 8.1	13.12
SiS_2	291213	14	3.76	7.3, 7.5, 7.7	27.64
SiS_2	291214	122	4.05	8.0, 8.0, 9.1	31.46
Sn_2S_3	653956	62	1.23	24.1, 29.2, 31.9	0
SnS	651018	62	1.26	40.5, 50.1, 64.0	0
SnS	30271	62	1.25	40.7, 50.3, 65.0	0.23
SnS	52108	62	1.24	40.6, 51.2, 64.6	0.29

Name	ICSD	Space group	E_g (eV)	Dielectric tensor	ΔE (meV/atom)
SnS	52110	62	1.24	41.1, 49.2, 65.9	0.3
SnS	43409	216	0.53	20.2, 20.2, 20.2	281.24
SnS_2	100611	164	2.32	9.0, 19.4, 19.4	0
SnS_2	43003	186	2.15	9.0, 18.8, 18.8	0.73
SnS_2	193391	227	1.96	14.4, 14.4, 14.4	4.38
SrS	651054	225	3.46	11.0, 11.0, 11.0	0
SrS	52111	221	2.72	23.2, 23.2, 23.2	299.74
SrS_2	642	140	2.35	9.0, 9.9, 9.9	0
TcS_2	81816	2	1.68	20.0, 22.0, 28.3	0
TiS_3	651177	11	0.98	11.1, 17.0, 20.2	0
Tl_2S	651245	146	1.25	32.9, 32.9, 35.8	0
Tl_2S_2	78161	140	0.98	22.2, 22.2, 53.2	0
Tl_2S_5	1911	19	2.4	18.4, 18.9, 19.7	0
Tl_4S_3	2647	14	1.25	35.8, 40.0, 40.4	0
TlS	651242	140	1	22.4, 22.4, 54.9	0
WS_2	202367	160	1.8	7.7, 14.0, 14.0	0
WS_2	651387	194	1.83	7.1, 14.0, 14.0	0.34
Y_2S_3	651408	11	2.4	14.2, 16.5, 16.6	0
Y_2S_3	651407	62	1.47	17.1, 17.3, 18.3	1.95
$Zn(S_2)$	651447	205	2.5	14.9, 14.9, 14.9	0
ZnS	291064	216	3.46	8.0, 8.0, 8.0	0
ZnS	107611	160	3.47	8.0, 8.0, 8.0	4.97
ZnS	291066	186	3.55	7.9, 7.9, 8.2	12.87
ZrS_2	601166	164	1.82	9.2, 50.8, 50.9	0
ZrS_3	604573	11	1.88	7.6, 11.2, 16.4	0

Table S6. Names, ICSD numbers, space groups, band gaps, dielectric tensors, and ΔE of binary chlorides

Name	ICSD	Space group	E_g (eV)	Dielectric tensor	ΔE (meV/atom)
$(ICl_3)_2$	24714	2	2.81	8.0, 10.1, 10.1	0
$(Si_5Cl_{12})(SiCl_4)$	2767	219	5.91	3.9, 3.9, 3.9	0
AgCl	56541	11	2.41	10.5, 10.6, 11.5	0

Name	ICSD	Space group	E_g (eV)	Dielectric tensor	ΔE (meV/atom)
AgCl	56539	225	2.41	11.0, 11.0, 11.0	1.84
$AlCl_3$	39566	12	6.73	3.7, 6.5, 6.5	0
$AsCl_3$	280796	19	4.89	5.0, 5.2, 29.0	0
Au_4Cl_8	201436	2	1.63	5.0, 13.3, 14.5	0
AuCl	6052	141	3.03	7.0, 7.0, 9.5	0
$AuCl_3$	22146	14	2.52	6.8, 7.4, 9.2	0
B_2Cl_4	14213	61	4.13	3.8, 4.8, 5.0	0
B_4Cl_4	27872	137	4.2	3.7, 4.0, 4.0	0
BCl_3	24526	176	6.22	2.6, 3.6, 3.6	0
$BaCl_2$	2191	225	6.72	6.8, 6.8, 6.8	0
$BaCl_2$	79891	14	6.45	8.3, 8.8, 8.9	26.06
$BaCl_2$	262674	62	6.37	7.8, 8.4, 8.7	32.83
$BaCl_2$	2190	189	5.82	8.6, 8.6, 9.9	34.79
$BeCl_2$	92583	72	7.81	3.0, 3.3, 5.0	0
$BeCl_2$	92586	142	7.89	4.0, 4.0, 4.2	6.23
$BiCl_3$	2866	33	4.46	18.7, 25.7, 114.2	0
BrCl	424850	36	3.04	3.0, 5.9, 58.1	0
$CaCl_2$	246416	58	7.05	6.1, 6.7, 7.9	0
$CaCl_2$	56769	60	7.06	6.5, 6.7, 7.5	8.49
$CaCl_2$	86209	62	7.06	4.8, 6.0, 6.5	17.71
$CdCl_2$	86440	166	4.87	4.3, 7.9, 7.9	0
$CeCl_3$	31575	176	3.88	7.5, 7.5, 9.6	0
CsCl	61515	225	6.11	5.3, 5.3, 5.3	0
CsCl	622366	221	6.35	7.1, 7.1, 7.1	49.66
$Ga(Ga_2Cl_7)$	67279	33	4.93	8.5, 11.2, 11.9	0
$Ga(GaCl_4)$	62664	52	4.78	11.0, 12.5, 14.2	0
$GeCl_4$	280880	14	5.46	3.9, 4.0, 4.1	0
$HfCl_4$	402054	13	5.38	4.6, 5.1, 9.1	0
Hg_2Cl_2	31173	139	3.59	11.6, 16.6, 16.6	0
$HgCl_2$	23277	62	4.22	9.8, 10.2, 16.0	0
ICl	411014	14	2.79	9.6, 11.6, 16.1	0
InCl	425449	63	1.89	25.3, 26.8, 54.6	0

Name	ICSD	Space group	E_g (eV)	Dielectric tensor	ΔE (meV/atom)
InCl	2432	36	1.89	25.2, 27.3, 59.5	0.1
$IrCl_3$	25716	70	3.38	5.0, 5.8, 6.7	0
$IrCl_3$	23171	12	3.27	4.9, 6.9, 6.9	14.24
KCl	165593	225	6.35	4.7, 4.7, 4.7	0
KCl	60402	221	6.27	6.0, 6.0, 6.0	83.7
$LaCl_3$	23146	176	5.77	7.1, 7.2, 9.0	0
LiCl	26909	225	7.73	9.9, 9.9, 9.9	0
$MgCl_2$	17063	164	6.98	3.0, 6.1, 6.1	0
$MgCl_2$	26157	166	6.92	3.7, 6.1, 6.1	0.65
$MoCl_3$	26108	12	1.54	9.1, 17.9, 139.1	0
$MoCl_3$	26109	15	2.13	5.0, 8.1, 9.0	28.08
$MoCl_5$	84620	2	0.87	16.4, 32.9, 33.0	0
NaCl	165592	225	6.43	5.1, 5.1, 5.1	0
NaCl	622368	221	5.34	11.2, 11.2, 11.2	162.8
Nb_3Cl_8	408645	164	1.07	4.9, 9.8, 9.8	0
$NbCl_4$	1010	12	2.02	5.3, 7.0, 13.2	0
$NbCl_5$	66537	14	3.43	6.7, 7.0, 7.0	0
$OsCl_4$	1165	65	2.1	21.9, 35.4, 63.3	0
$PbCl_2$	81976	62	4.83	24.6, 28.5, 30.9	0
$PbCl_2$	81978	14	4.82	24.8, 28.1, 31.4	0.24
$PbCl_4$	280975	15	2.74	8.2, 10.1, 12.4	0
$PdCl_2$	404624	148	2.85	9.1, 9.7, 9.9	0
$PdCl_2$	421221	14	2.28	5.9, 6.4, 9.9	8.78
$PdCl_2$	421220	10	2.46	4.0, 8.2, 10.4	26.17
$PdCl_2$	421213	58	2.52	4.6, 8.3, 10.4	27.23
$PtCl_2$	44512	58	2.46	5.0, 6.0, 9.6	0
$PtCl_3$	413423	148	1.91	10.3, 10.3, 11.2	0
RbCl	18016	225	6.12	4.7, 4.7, 4.7	0
RbCl	26877	221	6.08	6.5, 6.5, 6.5	59.84
Re_3Cl_9	14209	166	1.57	5.1, 5.7, 5.7	0
$ReCl_3$	62222	166	1.58	5.1, 5.7, 5.7	0
$ReCl_4$	10293	13	1.95	8.0, 16.0, 44.0	0

Name	ICSD	Space group	E_g (eV)	Dielectric tensor	ΔE (meV/atom)
$RhCl_3$	25764	12	3.07	5.7, 7.9, 7.9	0
$SbCl_3$	22191	62	4.68	7.8, 9.1, 73.3	0
$SbCl_5$	412110	14	2.9	5.4, 6.0, 8.0	0
Se_2Cl_2	37018	14	2.91	9.1, 15.9, 17.7	0
$SiCl_2$	85526	19	2.88	3.9, 4.1, 6.6	0
$SiCl_4$	62279	14	7.05	3.5, 3.5, 3.6	0
$SnCl_2$	81977	62	3.96	17.3, 53.1, 55.1	0
$SnCl_2$	81979	14	3.97	17.0, 52.9, 55.2	0.02
$SnCl_4$	411242	14	4.88	4.7, 4.9, 5.3	0
$SrCl_2$	28964	225	6.56	7.0, 7.0, 7.0	0
$TaCl_4$	402406	12	2.1	5.5, 6.1, 11.4	0
$TcCl_3$	261105	166	1.94	5.6, 6.4, 6.4	0
$TcCl_3$	262639	12	0.54	13.1, 27.3, 31.7	114.69
$TcCl_4$	26055	61	2.56	7.8, 8.1, 162.6	0
Te_3Cl_2	105	14	1.94	15.1, 29.0, 30.1	0
$TiCl_4$	280981	14	4.47	5.1, 5.8, 6.5	0
TlCl	61518	225	3.3	15.0, 15.0, 15.0	0
TlCl	109143	63	3.27	15.9, 16.9, 21.1	16.31
TlCl	29107	221	2.91	31.1, 31.1, 31.1	46.58
$TlTlCl_4$	4031	88	2.75	15.2, 15.7, 15.8	0
WCl_4	165263	12	1	11.6, 14.8, 18.7	0
WCl_6	425147	148	2.64	10.3, 10.3, 10.4	0
WCl_6	425148	164	2.54	8.6, 8.6, 12.8	0.03
Y_2Cl_3	23337	12	1.3	9.8, 9.8, 13.0	0
YCl_3	15684	12	5.96	5.0, 7.1, 7.1	0
$ZnCl_2$	27673	122	5.79	4.7, 4.7, 5.7	0
$ZnCl_2$	26152	137	5.57	3.8, 6.5, 6.5	7.57
$ZnCl_2$	26153	14	5.63	5.7, 12.3, 24.2	30.7
$ZrCl_2$	30052	160	1.48	4.4, 12.5, 12.5	0
$ZrCl_3$	43292	162	1.38	4.9, 11.2, 13.7	0
$ZrCl_4$	26049	13	4.97	5.0, 5.0, 10.2	0

Table S7. Names, ICSD numbers, space groups, band gaps, dielectric tensors, and ΔE of ternary fluorides.

Name	ICSD	Space group	E_g (eV)	Dielectric tensor	ΔE (meV/atom)
$((Kr_2F_3)(SbF_6))(KrF_2)$	279628	2	3.27	4.4, 5.4, 6.4	0
$(Ag_2C_2)(AgF)_8$	407646	81	3.29	15.9, 18.0, 18.3	0
$(AsCl_4)(AsF_6)$	33884	85	4.75	5.0, 5.0, 5.2	0
$(Br_3)(AsF_6)$	33811	2	2.94	5.7, 5.7, 8.4	0
$(BrF_2)_2(GeF_6)$	321	14	4.68	4.5, 4.7, 7.0	0
$(BrF_3)(AuF_3)$	93481	4	3.81	4.0, 4.3, 6.3	0
$(C_6F_5)Se(C_6F_5)$	410720	4	4.09	3.0, 3.0, 3.4	0
$(CF_2)_2Te_2$	73583	4	2.51	4.8, 8.7, 9.2	0
$(CF_3)(IF_4)$	401706	14	6.25	4.1, 4.3, 4.9	0
$(CF_3)_2O_3$	401780	2	6.96	2.0, 2.3, 2.6	0
$(CF_3)TeTe(CF_3)$	401990	14	3.5	4.1, 4.6, 4.6	0
$(ClF_2)(BiF_6)$	39555	2	3.88	5.0, 6.4, 8.9	0
$(CsF)(Br_2)$	69124	123	3.03	4.2, 4.2, 66.4	0
$(CsF)_2(Br_2)$	84021	139	3.82	5.1, 5.1, 13.4	0
$(H_3O)F$	24382	62	8.84	3.5, 5.5, 6.9	0
$(IF_4)(SbF_6)$	281154	73	6.17	3.8, 7.3, 7.3	0
$(IOF_2)(IO_2F_4)$	201202	14	3.95	4.6, 5.6, 5.7	0
$(InF_3)(SbF_5)_3$	421923	165	5.68	5.4, 5.8, 5.8	0
$(KrF)(AsF_6)$	279624	14	4.68	4.3, 4.5, 5.2	0
$(KrF)(BiF_6)$	279626	14	4.39	5.1, 5.3, 6.0	0
$(KrF)(SbF_6)$	279625	14	4.55	4.5, 4.6, 5.0	0
$(NH_4)(HF_2)$	415007	53	9.05	4.5, 5.9, 6.1	0
$(NH_4)F$	23766	186	7.81	3.7, 3.7, 3.7	0
$(NH_4)F(NH_3)$	419918	29	6.47	4.1, 4.4, 5.6	0
$(NO)F$	411510	19	5.11	4.6, 5.9, 19.0	0
$(PNF_2)_4$	31125	14	6.74	3.0, 3.3, 3.6	0
$(ReF_5(NF))$	33543	14	3.76	4.0, 4.2, 4.5	0
$(SeF_3)(NbF_6)$	9898	146	6.56	6.5, 6.5, 6.5	0
$(SnF)(AsF_6)$	816	155	4.53	9.4, 9.4, 14.3	0
$(TlF_3)(SbF_5)_3$	421924	147	3.92	4.7, 4.8, 4.8	0

Name	ICSD	Space group	E_g (eV)	Dielectric tensor	ΔE (meV/atom)
$Ag(AuF_4)_2$	85416	14	2.06	6.4, 11.2, 14.3	0
$Ag(BF_4)$	415320	62	5.3	5.5, 6.4, 19.7	0
$Ag(BiF_6)_2$	79879	2	2.45	9.0, 14.7, 16.0	0
$Ag(SbF_6)_2$	65186	2	2.81	5.2, 5.9, 6.1	0
$Ag(SnF_6)$	51505	2	2.66	6.6, 7.6, 9.4	0
$Ag(TaF_6)_2$	62543	2	3.19	6.1, 6.6, 7.1	0
$Ag(TiF_6)$	51506	2	2.32	20.7, 21.7, 23.1	0
$AgZnF_3$	28950	221	3.58	15.1, 15.1, 15.1	0
As_2F_7K	36332	14	6.2	6.5, 9.8, 14.7	0
$AsCl_2F_3$	25026	85	4.75	5.0, 5.0, 5.0	0
$AsCsF_4$	413041	4	5.94	5.3, 5.7, 6.9	0
$Au_3F_8(SbF_5)_2$	412235	14	2.11	5.0, 7.4, 7.8	0
$Ba(AlF_5)$	80565	4	9.47	9.5, 9.7, 10.0	13.53
$Ba(BF_4)_2$	240991	12	10.38	5.8, 6.2, 6.7	0
$Ba(BrF_4)_2$	428086	82	5.03	6.4, 8.9, 8.9	0
$Ba(SbF_5)$	68455	57	6.11	6.0, 6.2, 9.2	0
$Ba(SbF_6)_2$	39346	1	6.48	5.1, 5.5, 5.7	0
$Ba(ZnF_4)$	402926	36	7.02	9.7, 11.2, 13.0	0
$Ba(ZrF_6)$	1697	14	8.69	9.8, 11.9, 12.7	0
$Ba_{12}F_{19}Cl_5$	78943	189	7.46	7.1, 7.1, 8.0	0
$Ba_2(ZnF_6)$	21054	139	7.32	9.0, 10.7, 10.7	0
Ba_2NF	262049	166	1.96	21.3, 185.4, 185.4	0
Ba_2PdF_6	88802	64	3.93	6.4, 6.6, 6.6	0
Ba_2ZrF_8	85717	62	8.29	8.6, 9.5, 10.2	0
$Ba_3(In_2F_{12})$	48182	127	6.68	7.8, 10.3, 10.3	0
$Ba_7F_{12}Cl_2$	410679	174	7.6	8.0, 8.0, 8.7	0
$BaAlF_5$	37033	19	9.54	7.4, 7.7, 7.9	0
$BaAu_2F_8$	65289	82	4.52	5.3, 6.3, 6.3	0
$BaBeF_4$	414412	62	9.72	6.7, 6.7, 37.4	0
$BaFBr$	35393	129	6.11	7.6, 7.6, 8.0	0
$BaFI$	1128	129	5.02	8.0, 8.0, 9.5	0
$BaGaF_5$	200316	19	7.87	8.0, 8.1, 8.2	0

Name	ICSD	Space group	E_g (eV)	Dielectric tensor	ΔE (meV/atom)
$BaMgF_4$	182596	36	8.97	7.5, 8.0, 12.7	0
$BaPdF_4$	108991	140	3.62	6.0, 6.0, 6.0	0
$BaSnF_4$	166207	129	4.67	7.4, 23.9, 23.9	0
$BaSnF_6$	33788	148	7.27	7.9, 8.1, 8.1	0
$BaTeF_6$	88416	43	6.61	11.0, 11.1, 15.2	0
Bi_3NF_6	79395	57	3.9	18.5, 21.8, 27.1	0
$Bi_7F_{11}O_5$	167074	5	5.15	15.3, 26.4, 40.6	0
$BiLiF_4$	65404	88	6.26	10.6, 10.6, 17.8	0
CF_2Cl_2	33946	43	7.45	2.7, 3.4, 3.6	0
CF_3Cl	49696	36	8.43	2.5, 2.5, 3.0	0
CF_3I	73268	64	4.93	2.9, 3.8, 3.8	0
$CFCl_3$	74766	61	6.46	3.1, 3.1, 3.7	0
$Ca(AlF_5)$	69563	15	9.65	4.8, 6.0, 6.1	0.18
$Ca(HF_2)_2$	419144	70	9.9	4.0, 6.2, 8.3	0
$Ca(PdF_6)$	26164	148	4.28	5.2, 5.2, 5.9	0
$Ca(SiF_6)$	183914	148	9.81	4.8, 4.8, 5.2	0
$Ca(SnF_6)$	35723	148	7.7	5.7, 5.7, 6.0	0
Ca_2AlF_7	100308	62	9.36	6.1, 6.1, 8.1	0
$CaAlF_5$	171399	14	9.47	5.3, 6.2, 6.7	0
CaClF	1130	129	7.27	9.1, 9.1, 9.2	0
$CaPdF_4$	32674	140	3.29	6.0, 6.0, 6.7	0
$CaPtF_6$	37443	148	4.7	4.8, 4.8, 5.3	0
$CaZnF_4$	31366	88	7.77	10.6, 13.2, 13.2	0
$Cd(AuF_4)_2$	85413	124	3.72	4.6, 4.6, 5.2	0
$Cd(PdF_6)$	26166	148	3.86	5.7, 5.7, 6.6	0
$Cd(PtF_6)$	78906	148	4.2	5.1, 5.1, 5.8	0
Cd_4F_6O	74031	137	3.56	7.5, 8.3, 8.3	0
$CdSnF_6$	25017	148	6	6.5, 6.5, 6.9	0
$CdTiF_6$	16233	148	6.1	8.2, 8.2, 10.3	0
Ce_2SeF_4	21011	166	4.06	13.9, 13.9, 14.3	0
$CeZrF_7$	39776	4	4.37	7.3, 7.7, 7.8	0
ClF_2BF_4	202816	14	5.48	3.0, 4.2, 4.4	0

Name	ICSD	Space group	E_g (eV)	Dielectric tensor	ΔE (meV/atom)
ClF_2SbF_6	9899	2	5.26	4.4, 5.0, 7.6	0
$Cs(As_4F_{13})$	281641	82	6.78	8.6, 8.6, 12.2	0
$Cs(AsF_6)$	408070	148	7.55	4.4, 4.4, 4.7	0
$Cs(Au_2F_7)$	152057	15	3.77	4.3, 4.5, 5.3	0
$Cs(AuF_4)$	152056	71	4.46	4.8, 5.0, 5.8	0
$Cs(BF_4)$	21084	62	9.88	4.2, 4.4, 4.9	0
$Cs(Br_2F_7)$	426291	14	4.33	5.4, 5.6, 7.5	0
$Cs(BrF_4)$	426292	71	5.01	4.5, 7.2, 7.5	0
$Cs(BrF_6)$	65712	148	5.17	5.7, 5.7, 5.8	0
$Cs(HgF_3)$	15168	221	2.41	15.9, 15.9, 15.9	0
$Cs(PbF_3)$	93438	161	4.49	10.0, 11.5, 11.5	0
$Cs(Pd_2F_5)$	78777	74	2.42	13.0, 14.8, 146.6	0
$Cs(Sb_2F_7)$	14119	15	5.53	9.3, 10.1, 11.8	0
$Cs(SbF_6)$	201886	148	7.29	4.6, 4.6, 4.8	0
$Cs_2(BeF_4)$	23152	33	8.78	6.1, 6.8, 6.9	0
$Cs_2(GeF_6)$	35547	225	8.17	4.9, 4.9, 4.9	0
$Cs_2(NbF_6)$	72832	164	2.28	47.3, 259.7, 260.8	0
$Cs_2(PtF_6)$	78955	225	4.78	4.8, 4.8, 4.8	0
$Cs_2(SiF_6)$	38548	225	9.13	4.8, 4.8, 4.8	0
$Cs_2(TcF_6)$	425912	164	4.45	6.0, 6.0, 6.0	0
Cs_2CaF_4	82616	139	8.01	6.6, 9.5, 9.5	0
Cs_2HfF_6	25600	164	9.32	7.1, 7.2, 7.2	0
Cs_2HgF_4	72353	139	3.8	6.3, 17.2, 17.2	0
Cs_2PdF_6	28674	225	4.37	4.7, 4.7, 4.7	0
Cs_2PtF_6	35107	164	4.7	5.4, 5.5, 5.5	13
Cs_2SnF_6	291386	164	7.57	6.2, 6.2, 6.4	0
Cs_2ZrF_6	25598	164	9.06	7.3, 7.6, 7.6	0
$Cs_3(GeF_7)$	202917	127	6.27	7.5, 7.5, 8.1	0
$Cs_3(SiF_6)F$	9588	127	7.57	6.9, 6.9, 7.6	0
Cs_3TiF_7	9594	127	4.52	8.1, 8.1, 8.9	0
$Cs_4Mg_3F_{10}$	16084	64	8.31	6.1, 6.6, 10.2	0
$Cs_4Zn_3F_{10}$	71589	64	7.14	6.0, 6.0, 8.6	0

Name	ICSD	Space group	E_g (eV)	Dielectric tensor	ΔE (meV/atom)
$CsAlF_4$	10012	189	9.13	5.7, 5.7, 6.0	0
$CsBeF_3$	9870	62	8.73	4.6, 4.8, 5.6	0
$CsBiF_6$	15122	148	4.91	5.2, 5.2, 5.3	0
$CsCaF_3$	45309	221	9.15	8.6, 8.6, 8.6	0
$CsCdF_3$	49582	221	5.27	9.2, 9.2, 9.2	0
CsH_2F_3	415005	19	9.04	4.9, 5.0, 5.5	0
$CsHF_2$	45858	140	8.53	5.5, 6.0, 6.0	0
$CsLiF_2$	18020	15	8.5	6.6, 6.8, 8.1	0
$CsMgF_3$	49584	221	8.93	6.4, 6.4, 6.4	0
$CsNbF_6$	183851	148	7.12	5.9, 5.9, 6.5	0
$CsSbF_4$	201405	55	5.61	6.9, 7.1, 11.7	0
$CsSnF_3$	236903	14	4.7	7.1, 8.7, 21.3	0
$CsTeF_5$	200252	62	6.11	5.0, 6.3, 8.1	0
$F_2(OPOPO)F_2$	248122	54	7.53	2.0, 3.9, 4.2	0
F_5N_3W	201198	14	4.13	4.0, 4.7, 5.3	0
$F_6O_4Os_2$	240331	14	2.11	5.0, 7.0, 7.5	0
$F_6O_4Os_2$	240330	7	2.04	5.7, 7.5, 8.4	1.84
$F_7O_3Os_2$	240329	14	1.51	4.7, 6.3, 6.4	0
H_2F_3P	406359	36	8.42	3.2, 3.2, 7.0	0
$H_3OH_3F_4$	32569	2	9.59	3.3, 4.2, 8.3	0
H_3OHF_2	32568	14	9.21	3.8, 5.0, 8.8	0
HF_2N	404200	29	6.87	2.8, 3.0, 3.0	0
HOF	63681	19	5.97	3.1, 3.7, 5.7	0
$Hg((CF)(CF_2))_2$	165635	2	4.94	3.1, 3.5, 3.7	0
$Hg(AuF_4)_2$	85414	124	3.41	5.0, 5.0, 6.1	0
$Hg_3(AsF_6)_2$	9323	14	3.51	7.2, 7.5, 11.1	0
$Hg_3S_2F_2$	16927	199	3.15	18.5, 18.5, 18.5	0
Hg_4OF_6	99995	186	1.69	12.7, 367.7, 367.7	0
I_3AsF_6	15527	2	2.39	6.6, 8.6, 14.1	0
$I_4(SbF_6)_2$	63301	2	1.3	5.6, 9.0, 12.3	0
$I_5(AsF_6)$	59115	15	2.29	4.7, 8.2, 16.9	0
IO_2F	280804	19	4.16	14.8, 24.1, 25.0	0

Name	ICSD	Space group	E_g (eV)	Dielectric tensor	ΔE (meV/atom)
IOF_3	4076	19	4.76	6.9, 10.5, 12.0	0
$In(AsF_6)$	417952	148	4.46	13.3, 21.5, 21.5	0
$In(BF_4)$	50218	62	5.54	14.0, 14.3, 15.0	0
$K(AlF_4)$	166825	62	9.1	4.3, 4.7, 5.0	0
$K(AlF_4)$	60525	11	9.06	4.5, 4.7, 4.7	6.81
$K(AlF_4)$	285	123	9	8.0, 8.0, 8.8	14.39
$K(AsF_6)$	59413	148	7.27	4.5, 4.6, 4.6	0
$K(BF_4)$	21081	62	10.25	4.1, 4.3, 6.7	0
$K(BiF_4)$	63166	227	4.97	22.0, 22.0, 22.0	0
$K(BrF_4)$	10326	140	4.91	4.7, 4.7, 5.7	0
$K(CdF_3)$	201329	62	5.32	7.4, 7.5, 9.9	0
$K(CeF_4)$	23229	62	4.52	6.1, 6.6, 8.8	0
$K(GaF_4)$	203108	62	7.5	4.8, 5.2, 5.3	0
$K(HF_2)$	9345	140	9.15	4.1, 5.4, 5.4	0
$K(OsF_6)$	27664	148	3.6	5.2, 5.2, 5.2	0
$K(PF_6)$	25576	205	9.7	5.0, 5.0, 5.0	0
$K(Sb_2F_7)$	14118	14	5.31	6.7, 10.9, 25.6	0
$K(SbF_4)$	200572	59	5.67	6.5, 8.0, 8.7	0
$K(SnF_3)$	72472	2	4.39	7.0, 11.1, 13.0	0
$K(Te_2F_9)$	84364	2	5.94	6.0, 7.0, 9.8	0
$K(TeF_5)$	16155	57	6.73	4.0, 4.2, 5.2	0
$K(ZrF_5)$	83584	2	7.94	8.2, 8.8, 9.2	0
$K_2(AgF_4)$	421461	14	2.9	5.0, 5.5, 6.0	0
$K_2(AlF_5)$	81864	123	8.45	4.5, 4.5, 5.7	0
$K_2(BeF_4)$	50337	33	8.92	4.8, 6.8, 7.8	0
$K_2(BeF_4)$	153081	62	8.94	4.8, 6.7, 7.0	0.04
$K_2(BiF_5)$	418777	62	6.25	6.2, 6.4, 10.2	0
$K_2(GeF_6)$	24026	164	8	5.5, 5.5, 6.2	3.24
$K_2(HfF_6)$	47246	189	9	5.0, 5.0, 9.0	46.36
$K_2(PdF_4)$	33888	12	4.13	4.0, 4.7, 4.9	0
$K_2(ReF_6)$	1528	164	4.06	5.2, 6.3, 6.3	0
$K_2(SbF_5)$	39634	14	5.97	6.0, 7.0, 8.0	0

Name	ICSD	Space group	E_g (eV)	Dielectric tensor	ΔE (meV/atom)
$K_2(SiF_6)$	158483	186	9.51	5.0, 5.0, 5.3	0
$K_2(TcF_6)$	425914	164	4.34	5.5, 6.2, 6.2	0
$K_2(TiF_6)$	280318	164	6.51	6.1, 6.6, 6.6	0
$K_2(ZrF_6)$	865	15	7.88	5.7, 8.0, 9.0	0
K_2GeF_6	30310	186	8.08	5.4, 5.4, 5.4	0
K_2HfF_6	29514	15	8.52	5.5, 7.8, 8.1	0
K_2MgF_4	33519	139	8.53	4.0, 5.9, 5.9	0
K_2PdF_6	27486	164	4.2	5.2, 5.6, 5.6	0
K_2PtF_6	16892	164	4.61	4.8, 5.6, 5.6	0
K_2ReF_8	20029	62	3.26	4.8, 4.8, 5.5	0
K_2SiF_6	29407	225	9.43	4.6, 4.6, 4.6	5.44
K_2TaF_7	19067	14	7.54	4.9, 5.0, 5.4	0
K_2YF_5	20692	33	8.97	5.7, 5.9, 6.3	0
K_2ZnF_4	100298	139	6.59	4.7, 6.0, 6.0	0
$K_3(SiF_6)F$	23875	127	8.02	5.7, 7.2, 7.2	0
$K_3(Zn_2F_7)$	100299	139	6.5	5.1, 6.1, 6.1	0
K_3BeF_5	14114	130	8.27	5.4, 6.0, 6.0	0
K_3TaF_8	248063	186	5.52	5.4, 5.4, 5.5	0
K_3YF_6	416296	14	8.44	6.9, 7.4, 11.9	0
$KAuF_4$	10327	140	4.39	4.0, 4.0, 4.0	0
$KCaF_3$	153628	62	8.74	6.7, 6.9, 9.0	0
KH_3F_4	202107	167	9.52	4.6, 4.6, 11.4	0
$KMgF_3$	40477	221	9.15	5.8, 5.8, 5.8	0
KSb_4F_{13}	4049	82	5.96	14.6, 17.0, 17.0	0
$KTlF_4$	4046	144	4.08	6.8, 6.8, 9.1	0
KY_3F_{10}	427142	225	9.1	9.1, 9.1, 9.1	0
$KZnF_3$	44787	221	6.29	6.4, 6.4, 6.4	0
$La(Zr_2F_{11})$	424908	72	8.16	7.8, 8.4, 8.5	0
LaOF	76427	129	6.28	9.0, 13.6, 13.6	0
LaOF	30622	166	5.8	11.4, 12.4, 12.5	0.45
$LaRbF_4$	262425	62	8.85	6.0, 6.7, 8.2	0
LaSF	31938	129	2.45	13.0, 14.0, 14.0	0

Name	ICSD	Space group	E_g (eV)	Dielectric tensor	ΔE (meV/atom)
LaSeF	21010	194	2.66	15.1, 15.1, 17.6	0
$LaSnF_7$	78896	14	7.31	6.0, 6.8, 7.0	0
$Li(AsF_6)$	74831	148	7.52	4.6, 4.6, 5.1	0
$Li(AuF_4)$	33953	13	4.14	4.4, 4.8, 5.1	0
$Li(AuF_6)$	165209	148	3.96	4.8, 4.8, 5.5	0
$Li(HF_2)$	23883	166	10.38	4.6, 4.9, 8.6	0
$Li(InF_4)$	66693	60	6.23	7.4, 7.9, 11.7	0
$Li(NbF_6)$	165202	148	7.04	6.8, 6.8, 6.8	0
$Li(OsF_6)$	165214	148	3.51	5.2, 5.2, 5.6	0
$Li(PF_6)$	74830	148	10.13	4.5, 4.5, 4.8	0
$Li(RuF_6)$	165203	148	3.5	5.7, 5.7, 6.2	0
$Li(Sb_2F_7)$	428176	62	5.96	6.9, 23.7, 37.9	0
$Li(SbF_6)$	23924	148	7.16	4.8, 4.8, 5.0	0
$Li(TaF_6)$	165205	148	8.17	6.2, 6.2, 6.2	0
$Li_2(BeF_4)$	72422	148	9.9	3.9, 3.9, 4.2	0
$Li_2(GeF_6)$	69622	150	8.27	5.6, 5.8, 5.8	0
$Li_2(GeF_6)$	23406	136	7.97	5.2, 5.9, 5.9	3.38
$Li_2(NbF_6)$	201755	162	2.06	15.6, 126.7, 199.0	0
$Li_2(PdF_6)$	165212	136	4.04	5.5, 6.0, 6.0	0
$Li_2(PtF_6)$	165216	136	4.55	5.0, 5.4, 5.4	0
$Li_2(RuF_6)$	165210	136	2.48	30.9, 257.4, 257.4	0
$Li_2(ZrF_6)$	409667	14	8.54	7.3, 7.4, 10.0	10.53
$Li_2F(BF_4)$	426821	141	9.79	4.8, 4.8, 4.9	0
Li_2HfF_6	251074	162	9.64	7.4, 7.4, 9.4	0
Li_2SiF_6	425923	150	10.54	5.3, 5.5, 5.5	0
Li_2TiF_6	18313	136	6.19	6.8, 7.2, 7.2	0
Li_2ZrF_6	155020	15	8.93	8.3, 9.4, 10.4	0
Li_2ZrF_6	2644	162	8.93	7.8, 7.8, 10.0	0.69
$Li_3(AlF_6)$	34672	33	10.31	6.3, 6.7, 7.1	0
$LiAuF_4$	9908	15	4.18	4.3, 4.5, 5.1	3.06
$LiBF_4$	171375	152	10.61	3.6, 4.9, 5.0	0
$LiBaF_3$	45310	221	8.63	11.3, 11.3, 11.3	0

Name	ICSD	Space group	E_g (eV)	Dielectric tensor	ΔE (meV/atom)
$LiBiF_6$	15119	148	4.5	6.0, 6.1, 6.1	0
$LiSbF_4$	428177	198	6.58	21.9, 21.9, 21.9	0
$LiYF_4$	73709	88	10.13	7.6, 7.7, 7.7	0.53
$Mg(PdF_6)$	26163	148	3.95	4.8, 4.8, 5.5	0
Mg_2NF	262327	141	3.39	8.7, 8.7, 13.3	0
Mg_3NF_3	262325	221	5.09	7.7, 7.7, 7.7	0
$MgAu_2F_8$	65287	14	3.94	4.0, 4.2, 5.0	0
$MgPbF_6$	15106	148	5.05	6.7, 6.7, 7.0	0
$MoOF_4$	16867	14	5.17	3.9, 4.5, 6.2	0
$N_3S_3F_3$	21015	148	5.56	4.7, 5.2, 5.2	0
$N_4S_4F_4$	71039	114	4.87	3.8, 5.1, 5.1	0
$NH_4(F(HF)_3)$	38337	161	9.51	4.9, 4.9, 7.0	0
$NH_4(F(HF)_4)$	38338	88	9.63	3.0, 4.2, 4.2	0
NPF_2	9684	36	7.24	2.8, 3.3, 5.5	0
$Na(AlF_4)$	248088	63	8.96	4.4, 4.8, 8.7	0
$Na(AsF_6)$	184562	148	7.53	3.0, 3.5, 3.5	0
$Na(BF_4)$	161160	63	10.19	3.3, 4.0, 4.1	0
$Na(HF_2)$	415006	166	9.14	2.8, 2.8, 5.5	0
$Na(MgF_3)$	158931	62	9.03	5.3, 5.3, 9.5	0
$Na(Sb_3F_{10})$	1968	173	6.32	6.5, 17.4, 17.4	0
$Na(SbF_4)$	24750	14	5.64	6.5, 7.6, 8.0	0
$Na(TiF_4)$	389	60	2.95	8.6, 67.7, 230.0	0
$Na_2(BeF_4)$	12101	62	9.02	3.5, 3.7, 3.7	0
$Na_2(BeF_4)$	28105	14	9.06	3.8, 4.0, 4.4	6.82
$Na_2(GeF_6)$	69623	150	8.02	4.4, 4.4, 5.4	0
$Na_2(PtF_6)$	428575	150	4.77	4.2, 4.2, 5.3	0
$Na_2(SbF_5)$	28061	19	6.21	5.4, 5.8, 7.0	0
$Na_2(SiF_6)$	40917	1	9.46	4.2, 4.2, 5.5	0
$Na_2(SiF_6)$	61274	150	9.31	4.2, 4.2, 5.4	20.23
$Na_2(TiF_6)$	40916	1	6.6	5.2, 5.2, 8.6	0
Na_2AgF_4	425149	14	2.85	5.1, 5.2, 5.8	0
$Na_3(AlF_6)$	74200	14	9.14	4.0, 4.9, 8.4	0

Name	ICSD	Space group	E_g (eV)	Dielectric tensor	ΔE (meV/atom)
$Na_3(TaF_8)$	260875	15	6.82	4.7, 8.0, 10.0	0
$Na_5(F(ZrF_6)_2)$	155759	12	8.44	5.7, 6.5, 6.8	0
$Na_5Hf_2F_{13}$	251075	12	8.87	5.4, 6.1, 6.4	0
$NaAgF_4$	9903	140	3.34	5.1, 5.1, 5.3	0
$NaAuF_4$	9905	140	4.05	4.7, 4.7, 5.1	0
$NaBiF_6$	15120	148	4.89	4.5, 5.0, 5.0	0
$NaMgF_3$	156157	63	8.88	4.0, 4.7, 7.5	33.07
$NaZnF_3$	72320	62	6.36	5.8, 5.9, 8.9	0
$NbCl_4F$	26155	2	3.63	6.0, 7.0, 7.1	0
$NbSbF_{10}$	16095	2	6.44	5.3, 7.4, 7.7	0
$O_2(AuF_6)$	411864	2	1.39	14.8, 17.3, 21.4	0
OsO_3F_2	73732	14	3.49	4.3, 6.2, 9.4	0
$OsOF_4$	417246	14	2.45	5.9, 6.1, 6.8	0
$OsOF_4$	417245	19	2.39	5.7, 6.9, 7.9	4.1
$OsOF_5$	16872	62	2.71	5.0, 5.1, 5.7	0
$OsOF_5$	240332	33	2.81	5.0, 5.0, 5.3	0.34
PHF_2	406360	19	4.6	6.2, 7.6, 15.2	0
PHF_4	406358	14	9.58	3.0, 3.0, 3.1	0
POF_3	250498	164	7.73	2.4, 3.5, 3.5	0
Pb_2F_2O	10416	137	3.88	14.8, 14.8, 18.0	0
Pb_2RhF_7	37141	14	3.85	12.5, 14.8, 16.3	0
PbBrF	30288	129	3.59	21.1, 33.0, 33.0	0
PbClF	30287	129	4.64	19.0, 28.3, 28.3	0
$PbF(AsF_6)$	411788	2	5.69	11.1, 11.7, 20.1	0
$PbF(SbF_6)$	429016	2	5.12	10.6, 13.3, 25.6	0
PbIF	56667	129	2.28	25.0, 38.5, 38.5	0
$PbPdF_4$	108992	140	3.01	8.2, 11.3, 11.3	0
$Pd(AuF_4)_2$	50213	14	2.95	5.2, 10.6, 12.0	0
$Pd(ZrF_6)$	73133	148	3.99	7.1, 7.1, 7.9	0
$PdPtF_6$	64661	148	3.06	8.6, 8.6, 10.4	0
$Pt(PF_3)_4$	418726	217	6.28	3.7, 3.7, 3.7	0
$Rb(AlF_4)$	54120	127	9.16	5.8, 5.8, 6.2	0

Name	ICSD	Space group	E_g (eV)	Dielectric tensor	Δ*E* (meV/atom)
$Rb(AlF_4)$	54123	59	9.2	5.7, 6.0, 6.1	1.03
$Rb(AlF_4)$	54119	123	9.08	6.2, 6.2, 6.9	2.22
$Rb(AsF_6)$	408069	148	7.38	4.2, 4.2, 4.3	0
$Rb(BF_4)$	21083	62	10.05	3.9, 4.0, 5.8	0
$Rb(Be_2F_5)$	28541	1	9.15	4.2, 4.2, 4.5	0
$Rb(BrF_4)$	65713	140	5.05	5.6, 5.6, 12.4	0
$Rb(HF_2)$	45859	140	8.87	4.4, 5.5, 5.5	0
$Rb(SF_5)$	65221	62	6.33	4.1, 6.7, 7.6	0
$Rb(SbF_6)$	408071	148	7.15	4.6, 4.6, 4.8	0
$Rb(ZnF_3)$	41618	194	6.72	5.9, 5.9, 6.4	0
$Rb_2(GeF_6)$	68982	225	8.15	4.7, 4.7, 4.7	0
$Rb_2(GeF_6)$	25662	186	8.07	5.2, 5.2, 5.3	3.36
$Rb_2(HfF_6)$	25599	164	9.25	6.9, 7.1, 7.1	0
$Rb_2(NbF_6)$	72831	164	2.27	103.6, 175.7, 199.7	0
$Rb_2(PdF_6)$	28675	225	4.41	4.6, 4.6, 4.6	0
$Rb_2(SiF_6)$	38547	225	9.25	4.4, 4.4, 4.4	0
$Rb_2(TcF_6)$	425918	164	4.41	5.0, 5.0, 5.4	0
Rb_2BeF_4	61800	33	8.71	5.1, 6.0, 6.1	0
Rb_2GeF_6	26633	164	7.99	5.2, 5.2, 5.9	9.65
Rb_2MgF_4	69681	139	8.33	5.0, 5.6, 5.6	0
Rb_2PtF_6	35108	164	4.68	4.8, 5.2, 5.2	0
Rb_2ZrF_6	25597	164	9	7.2, 7.6, 7.6	0
Rb_3SiF_7	9589	127	7.69	5.8, 6.7, 6.7	0
Rb_3TiF_7	9595	127	4.6	7.5, 7.8, 7.8	0
$Rb_6Mg_6F_{18}$	410385	194	8.93	5.5, 5.5, 5.8	0
$RbAuF_4$	9907	140	4.49	4.0, 4.3, 4.3	0
$RbBiF_6$	15121	148	4.78	5.0, 5.0, 5.3	0
$RbCaF_3$	201252	221	8.46	13.8, 13.8, 13.8	0
$RbCdF_3$	49587	221	5.14	16.3, 16.3, 16.3	0
$RbHfF_5$	95846	14	8.82	6.6, 7.6, 8.3	0
$RbMgF_3$	49585	221	9.18	5.6, 5.6, 5.6	0
$RbMgF_3$	33689	194	8.85	5.5, 5.5, 5.8	1.32

Name	ICSD	Space group	E_g (eV)	Dielectric tensor	ΔE (meV/atom)
$RbPbF_3$	161148	62	5.34	8.0, 9.0, 12.2	0
$RbPd_2F_5$	72299	74	2.43	9.0, 13.4, 24.6	0
$RbSb_2F_7$	200574	14	5.27	8.0, 11.3, 20.3	0
ReF_3O_2	415424	64	4.86	4.3, 4.5, 5.3	0
ReF_3O_2	415421	14	4.86	4.0, 4.9, 8.2	3.27
$ReFO_3$	415418	13	4.37	4.0, 4.6, 12.1	0
$RuOF_4$	417249	14	2.94	17.8, 23.5, 23.8	0
$RuOF_4$	417248	19	2.35	7.2, 9.9, 13.2	0.17
S_4N_5F	38254	2	3.57	6.0, 6.2, 7.3	0
SOF_2	48148	14	6.99	2.8, 3.4, 4.7	0
$SbCl_3F_2$	380014	82	3.07	4.1, 6.8, 6.8	0
$SbCl_3F_2$	200039	79	2.97	3.8, 6.2, 6.2	4.23
$SbCl_4F$	74783	82	2.65	5.6, 5.8, 5.8	0
SbOF	21099	61	4.45	12.9, 14.2, 21.1	0
SbOF	19019	62	4.32	9.1, 9.7, 32.1	4.28
$SeOF_2$	12110	29	5.4	4.4, 6.8, 7.9	0
SiH_3F	60065	14	7.36	4.1, 4.6, 6.4	0
$Sn(SnOF_5)$	409393	12	2.94	9.6, 12.4, 14.2	0
$Sn_2(SnOF_2)_2$	948	12	3.48	16.2, 18.3, 22.0	0
Sn_2ClF_3	200032	19	4.57	26.0, 26.5, 26.5	0
$Sn_2F_3BF_4$	15263	14	5.43	8.1, 8.7, 22.0	0
Sn_2F_3Cl	2088	198	4.57	26.7, 26.7, 26.7	0.26
Sn_2F_3I	2419	20	3.12	16.9, 17.6, 46.4	0.22
Sn_2IF_3	38268	63	3.12	16.9, 17.6, 46.4	0
Sn_3BrF_5	200031	14	3.95	43.0, 43.2, 69.7	0
Sn_4OF_6	78356	19	4.2	13.7, 14.2, 17.9	0
SnClF	647	62	4.34	14.1, 18.6, 48.6	0
$Sr(BeF_4)$	404396	14	9.78	5.9, 6.0, 6.9	0
$Sr(SbF_5)$	68454	57	6.14	6.0, 6.6, 12.6	0
$Sr(TaF_7)$	417254	11	7.49	7.6, 8.0, 8.2	0
Sr_2InF_7	38307	14	6.99	7.0, 7.2, 8.8	0
SrClF	68373	129	7.36	7.4, 7.6, 7.6	0

Name	ICSD	Space group	E_g (eV)	Dielectric tensor	ΔE (meV/atom)
SrFBr	35392	129	6.32	7.6, 7.6, 7.9	0
$SrMgF_4$	193584	36	8.85	6.4, 7.3, 15.3	0
$SrPdF_4$	108990	140	3.45	5.7, 5.7, 6.0	0
$SrZnF_4$	31367	88	7.97	7.4, 13.1, 13.1	0
$TaCl_4F$	27413	82	4.24	5.2, 6.2, 6.2	0
TcO_3F	249509	14	3.89	4.8, 7.2, 11.2	0
$Te(OTeF_5)_6$	2174	148	4.03	4.2, 4.3, 4.3	0
$Te_2O_3F_2$	82162	2	4.62	15.6, 22.8, 31.5	0
$TeOF_2$	88415	4	5.63	12.8, 19.0, 20.4	0
$Tl(AlF_4)$	202458	15	5.76	9.0, 10.9, 62.1	0
$Tl(AsF_6)$	417954	148	5.91	8.0, 8.0, 8.4	0
$Tl(BF_4)$	300222	62	7	7.5, 7.7, 10.4	0
$Tl_2(AlF_5)$	109365	63	5.11	10.6, 10.9, 29.2	0
$Tl_2(SiF_6)$	38549	225	6.25	11.1, 11.1, 11.1	0
$Tl_2(SnF_6)$	410801	164	5.57	16.3, 23.9, 23.9	0
$Tl_2(TiF_6)$	410802	164	4.54	14.9, 17.6, 17.6	0
Tl_2AlF_5	25616	20	5.16	10.1, 10.5, 25.0	0.62
Tl_2BeF_4	171183	62	5.65	15.7, 19.7, 20.0	0
$TlPF_6$	28899	205	7.65	8.0, 8.0, 8.0	0
$TlSbF_4$	201084	14	5.14	14.0, 16.9, 22.9	0
$TlSnF_7$	78899	14	3.4	6.3, 7.7, 8.4	0
$TlTeF_5$	90619	62	5.77	9.6, 10.3, 13.8	0
WSF_4	249977	29	4.22	4.0, 5.0, 6.1	0
YFS	250885	129	2.24	12.0, 15.5, 15.5	0
YFSe	2635	62	2.53	10.6, 13.1, 21.9	0
$YLiF_4$	96728	15	10.13	7.0, 7.7, 7.7	0
YOF	184004	166	6.62	11.3, 12.0, 12.0	0
YOF	76426	129	6.57	9.0, 14.7, 14.7	6.61
$YSnF_7$	74963	14	7.16	6.0, 6.7, 6.9	0
$Zn(PdF_6)$	26165	148	3.66	5.8, 5.8, 6.7	0
$ZnPbF_6$	15107	148	3.46	7.9, 7.9, 8.7	0
$ZnPtF_6$	37444	148	4.18	5.1, 5.1, 5.8	0

Name	ICSD	Space group	E_g (eV)	Dielectric tensor	ΔE (meV/atom)
$ZnSnF_6$	25012	148	5.85	6.2, 6.2, 6.9	0
